# A Dietary Supplement Containing Nucleotides, Oligosaccharides, Vitamin E and β-Carotene Promotes Immune Response and Gut Microbiota Changes in Kittens

**DOI:** 10.3390/ani15233504

**Published:** 2025-12-04

**Authors:** Willy Joly, Matthew Harrison, Jeremy Laxalde, Virginie Gaillard

**Affiliations:** 1 Royal Canin Research Center, 650 Avenue de la Petite Camargue, 30470 Aimargues, France; 2 Waltham Petcare Science Institute, Mars Petcare, Waltham on the Wolds, Melton Mowbray, Leicestershire LE14 4RT, UK

**Keywords:** cytokines, feline, fructooligosaccharides, immune, kitten, microbiota, nucleotides, vaccination, xylooligosaccharides

## Abstract

Kittens are born with some maternally derived antibodies (MDA) that, together with maternal gut microbiota, bring some form of early defense against pathogens. However, this protection declines and the rapid changes and challenges the kitten typically encounters can adversely affect their health. Nutrition can modulate the immune system and gut microbiota, offering a boost to protection following weaning. This controlled, randomized, double-blinded study tests a dietary supplement containing nucleotides, oligosaccharides, vitamin E and β-carotene in 50 domestic short-haired kittens. Control kittens were fed standard, dry kitten diet whilst test kittens received the same diet with supplement for 52 weeks. Kittens received routine vaccinations and blood samples were collected throughout the study for standard hematology, serum antibodies and cytokine expression. Rectal fecal samples were analyzed for gut microbiota via 16S rRNA gene sequencing. Supplemented kittens showed significantly improved antibody response to *Chlamydia* vaccine at various timepoints versus controls, with no consistent effects of supplementation on responses to other vaccines. Several cytokines were significantly influenced by the supplement, which also significantly altered gut microbiota diversity. In conclusion, a dietary supplement was shown to promote immune response and gut microbiota changes in kittens and may help to support a healthy transition into adulthood.

## Simple Summary

Kittens are born with some protection against pathogens and continue to receive this protection via their mother’s milk. They also receive a starter community of healthy gut microbiota from their mother, which offers defense against ingested harmful bacteria. Following weaning, this protection can start to diminish, leaving kittens vulnerable to infections. Certain nutrients and ingredients are known to help boost these immune defenses. This study tested the effects of feeding a diet with or without a nutritional supplement to 50 kittens for 52 weeks. All kittens received routine vaccinations against five common diseases and were tested throughout the study for immune responses. Their gut microbiota were also examined regularly via analysis of feces. The dietary supplement improved the kittens’ immune response to one of the five vaccinations and increased levels of several immune markers. Kittens receiving the supplement also showed beneficial changes to their gut microbiota. In conclusion, the dietary supplement tested here improved antibody response to *Chlamydia* vaccination and altered cytokine profiles. This supplement could be a beneficial addition to kitten diets to enable a healthy transition to adulthood.

## 1. Introduction

The immune system is a complex network of organs, tissues, cells and proteins largely involved in defending the body against infection. This is achieved via the innate and adaptive immune cells which destroy antigens and produce pathogen-specific antibodies [1]. Although mammals are born with some maternally derived antibodies (MDA) which also continue to be transferred during lactation, the protection these offer declines over time [2]. Another line of defense in the newborn is the community of gut microbiota gained in utero. The gut microbiota profile changes rapidly in kittens during the first month of life and evidence indicates post-weaning stabilization from eight weeks [3]. An immature immune system, together with the various stresses and changes a young animal typically encounters, can potentially have adverse effects on their health.

Nutrition can modulate the immune system, subsequently exerting effects on health [4]. Nutrients can impact immune cells both directly and indirectly, inducing changes in function or acting via the gut microbiota [5]. There is evidence to suggest that nucleotides, oligosaccharides, phytochemicals and vitamins can exert beneficial effects on the immune system.

Nucleotides form the basic constituents of nucleic acids and play a role in almost all biological processes in the body including energy transfer, intracellular signaling and protein synthesis. Although nucleotides are mainly produced endogenously, exogenous sources have been shown to be conditionally essential during growth and development [6]. Nucleotides have been found in companion animal colostrum and milk [7]; supplementation with dietary nucleotides mimicking those in the mother’s milk has been reported to improve immune response capacity of puppies at weaning [8]. Moreover, in mice, dietary nucleotides have been shown to have a positive impact on the maturation and differentiation of intestinal lymphocytes; this leads to faster development of antigen-specific markers on T- and B-cells which are key for antigen presentation and T-cell priming [9].

Fructooligosaccharides (FOS) are non-digestible, soluble plant sugars consisting of fructose oligomers and a glucose molecule, attached via β-(2−1) glycosidic linkages. FOS are prebiotics shown in cats to selectively promote growth of certain health-associated colonic bacteria such as *Bifidobacteria* and decrease *Escherichia coli* populations [10]. The immunomodulatory and anti-inflammatory actions of *Bifidobacteria* have been described [11]. Short-chain FOS (scFOS) are a sub-category of FOS with a reduced degree of polymerization (3−-5). Whilst evidence describing the benefits of scFOS on the immune system of companion animals is scarce, feeding scFOS to pregnant Beagles was shown to increase IgG concentrations in colostrum and milk [12].

Xylooligosaccharides (XOS) are non-digestible, soluble plant sugars consisting of a xylose backbone, linked by β-(1−-4) xylosidic bonds [13]. Like FOS, XOS has known prebiotic properties, able to stimulate intestinal growth of beneficial *Lactobacilli* and *Bifidobacteria* [14,15]. XOS has been shown to enhance the proliferative response of lymphocytes [16] and down-regulate the inflammatory response in vitro [17].

Vitamin E is an essential nutrient and is the major lipid-soluble component of the antioxidant defense system. When fed at supplemental levels, this vitamin has been shown to enhance lymphocyte functionality in cats [18].

β-carotene is a natural pigment synthesized by plants, with proven antioxidant activity in vitro and in animal models. The absorption of β-carotene by cats and dogs has been shown and its enhancement of both cell-mediated and humoral immune responses in dogs demonstrated [19,20].

A previous study reported strengthening of the humoral immune response to vaccination in domestic short-haired kittens fed a nutrient cocktail containing nucleotides, scFOS, XOS, β-carotene and vitamin E for 28 weeks [21]. The current study examines the effects of the same cocktail on immune function and gut microbiota in domestic short-haired kittens over a period of 52 weeks. As a result of supplementation, we hypothesize (1) enhancement of immune function measures such as *Chlamydia* antibody titers and (2) a positive shift in gut microbiota according to measures such as the Shannon index.

## 2. Materials and Methods

### 2.1. Study Design

This was a randomized, double-blinded prospective study using a parallel, matched-group design.

### 2.2. Animals and Diets

Fifty domestic short-haired specific-pathogen-free (SPF) kittens from 27 litters were randomized to control group (group 1; n = 25) or test group (group 2; n = 25), balanced for sex, genetics (through equal distribution of kittens from the same litter across groups) and weight. Kittens were identified via a subcutaneous transponder and were also numbered 1 to 50 for the study. Kittens were progressively weaned onto solid food from 4 weeks of age and were fully weaned onto their respective diets by 8 weeks of age. Control kittens received an extruded dry, specifically formulated experimental diet (Royal Canin^®^, Aimargues, France), also fed to the mothers throughout gestation until weaning of their kittens. Test kittens received the same diet supplemented with 3.3 g yeast extract rich in nucleotides (PetMOD, Prosol, Madone, Italy), 4.5 g scFOS (Profeed, Tereos, Moussy-le-Vieux, France), 3 g XOS (XOS 35, Longlive, Qingdao, China) and 7 mg β-carotene per kg of diet. The test diet contained 471 mg vitamin E, whereas control diets contained 214 and 247 mg vitamin E for the first (1) and second batch (2), respectively. Control and test diets were formulated from the same base recipe consisting of poultry protein (dried), animal fat, maize flour, rice, vegetable protein isolate, animal protein (hydrolyzed), fish oil, soya oil, beet pulp and vegetable fibers. Nutrient analysis of the diets was performed post-production at Eurofins Ltd. (Wolverhampton, UK; Table 1). Diets were nutritionally complete and balanced according to the Association of American Feed Control Officials (AAFCO) and complied with nutritional guidelines set out by the European Pet Food Industry (FEDIAF) and National Research Council (NRC) 2006 [22]. Diets were stored in a climate-controlled room and kept in sealed containers once opened.

### 2.3. Ethics Approval Statement

The study was conducted in accordance with the Mars Animal Research Policy (www.mars.com), accessed on 1 October 2025, adhering to the 3Rs approach to animal research, as previously described [23]. The study complied with ARRIVE guidelines [24] and was carried out in accordance with EU Directive 2010/63/EU for animal experiments. All protocols were approved by the Royal Canin Ethic Committee (protocol n°170518-21 validated the 14 June 2018 and protocol n°170518-21A validated the 22 January 2019), the Ethics and Animal Experimentation Committee at Isoquimen S. L. and Department of Territory and Sustainability, Directorate General of Environment Policy of the Generalitat de Catalunya (Autonomous Govern of Catalonia) (procedure registered with the order number: 10416). No animal harm was caused due to the study.

### 2.4. Housing, Husbandry and Veterinary Care

The study was conducted at Isoquimen S. L. (Barcelona, Spain). Until 8 weeks of age, kittens were housed with their mothers in SPF conditions and then transferred into separate rooms under controlled conditions, complying with regulations on animal handling, room cleaning and health requirements for the staff. Kittens were socialized from birth to 8 weeks of age and from 8 weeks of age to adulthood according to standard operating procedures of the facility. Housing temperature was maintained at 15–24 °C and humidity ranged from 30 to 90% during the study. Artificial lighting followed a 12 h light/12 h dark cycle from 6 am to 6 pm. The air was ventilated with positive pressure. Rooms were enriched with shelves, cat trees, scratching boards, barrels, beds and toys.

From 8 weeks of age, kittens from each dietary group were housed in 14 m^2^ rooms, separated by gender. Females (10 per group) were gathered in two rooms and males (15 per group) in two other rooms. Kittens had ad libitum access to water through water dispensers and were offered their respective diets ad libitum twice daily, once in the morning and once in the evening.

General observations were carried out once daily and any abnormal symptoms or behaviors recorded. Body weights were recorded weekly (kg) on a calibrated digital weighing platform according to the standard operating procedure of the facility. To monitor growth, front leg length and thoracic circumference measurements were recorded weekly for each kitten from 12 to 27 weeks of age and thereafter monthly. Regular physical examinations were performed by a veterinarian throughout the study. These included assessment of general health, body condition, hair coat and skin, ears, mouth, nose, throat, musculoskeletal system, eyes, abdomen, external urogenital system, anal and perianal region, limbs, temperature and behavior, as well as auscultation of the heart and lungs and abdominal palpation. Kittens in the control group were neutered at 39 weeks (female) and 32 weeks (male). Kittens in the supplemented group were neutered at 28 weeks (female) and 26 weeks (male). Variations in age at neutering were due to facility constraints.

### 2.5. Vaccination Schedule

A routine preventative medication schedule was followed, and kittens received a PUREVAX^®^ RCPCh (Boehringer Ingelheim, Duluth, GA, USA) vaccine containing attenuated feline viral rhinotracheitis (strain feline herpes virus (FHV) F2), inactivated feline calicivirus (strains FCV 431 and G1), attenuated feline *Chlamydia* (strain 905) and attenuated feline panleukopenia virus (FPV; strain PLI IV) at weeks 8, 12 and 52. At weeks 12 and 52, they also received a Eurican R (Boehringer Ingelheim, Duluth, GA, USA) vaccination containing inactivated rabies virus.

### 2.6. Blood Sample Collection

Jugular blood samples were collected at 4, 8, 10, 12, 14, 16, 18, 20, 28, 36, 44 and 52 weeks of age with 25G needles. Blood volumes sampled were adjusted according to age and weight of the kittens.

### 2.7. Standard Hematology Analysis

Blood for standard hematology analysis was collected in tubes containing EDTA and stored at 4 °C until analysis the same day. Serum samples were collected and placed into a gel serum tube until clotting and then were centrifuged at 4500 rpm for 10 min at 4 °C ± 2 °C to obtain serum. Serum samples were divided into aliquots and stored at −20 °C until analysis. Week 4 samples were analyzed using an automated blood cell analyzer (ADVIA 120 Hematology Analyzer; Siemens Healthcare Diagnostics Inc., Tarrytown, NY, USA) and the remaining samples analyzed using a Cell-DYN^®^ 3700 Hematology Analyzer (Abbott, Chicago, IL, USA). The following hematological parameters were determined: white blood cells (total and differential counts), red blood cells (total count), hematocrit, platelet count, Mean Corpuscular Hemoglobin (MCH), Mean Corpuscular Hemoglobin Concentration (MCHC) and Mean Corpuscular Volume (MCV).

### 2.8. Serum Antibodies

Antibodies against feline calicivirus (FCV antibody EIA 96 well plate, EIA F1008-AB02, EVL, serum dilutions ranging from 1/50 to 1/350), feline herpes virus (FHV antibody EIA 96 well plate, EIA F1007-AB02, EVL, serum dilutions ranging from 1/50 to 1/400), feline *Chlamydia* (ab EIA, F1009-AB01, EVL, serum dilutions ranging from 1/50 to 1/800), panleukopenia (feline parvo virus ab EIA, F1004-AB01, EVL, serum dilutions ranging from 1/300 to 1/8100) and rabies virus (Platelia Rabies II Kit ad usum vet, Biorad, ref 3550180, sera diluted 1/100) were measured via ELISA kits in the sera sampled from 8 weeks to 52 weeks of age [21]. Samples were analyzed by VetAgro Sup (Laboratoire Leptospires et Analyses Veterinaires, Marcy L’Etoile, France). The samples were processed as previously described in Atwal et al., 2023 [21].

### 2.9. Measurements of Cytokine Expression via Whole Blood Assay

For cytokine expression analysis, 500 μL heparinized (8–10 IU heparin/mL) whole blood samples were diluted 1:1 in R-10 culture medium (1% RPMI 1640, 100 μg/mL penicillin, 100 μg/mL streptomycin; Gibco, Life Technologies, Grand Island, NY, USA, ref. 15140) in a sterile Falcon tube and mixed. In total, 100 μL of each diluted blood sample was pipetted into 96-well flat-bottomed microtiter plates with lids (Thermo Fisher Scientific, Suzhou, China, ref. 167008) and exposed to three different conditions by adding 100 μL R-10 medium (unstimulated, negative control), 100 μL of a 10 μg/mL solution of phytohemagglutinin (PHA; positive control; Sigma-Aldrich, Saint Louis, MO, USA, ref. L1668) or 100 μL of a 10 μg/mL solution of rabies antigen (RA; Creative Diagnostics, Shirley, NY, USA, ref. DAGF-021). Each condition was duplicated for each sample. Plates were cultured for 48 h at 37 °C in a humidified incubator under 5% CO_2_ atmosphere conditions. Culture supernatants were harvested at 48 h by centrifuging at 400 *g* for 10 min and immediately stored in 96-well plates at −80° C until analysis. Culture supernatants were assayed for cytokine expression via Luminex XMAP^®^-based technology (MILLIPLEX^®^ MAP Feline Cytokine/Chemokine Magnetic Bead Panel, Merck, Darmstadt, Germany, ref. FCYTMAG-20K-PMX, batch 3126519), using a 19-plex premixed kit to be used for the simultaneous quantification of the following analytes: sFas, Flt-3L, GM-CSF, IFN-γ, IL-1β, IL-2, IL-4, IL-6, IL-8, IL-12 (p40), IL-13, IL-18, KC, MCP-1, PDGF-BB, RANTES, SCF, SDF-1 and TNF-α. IL-10 expression in supernatants was determined using a Feline IL-10 ELISA kit (Sigma-Aldrich, Saint Louis, MO, USA, ref. RAB0590-1KT, batch 1129F0954).

Quantification of each cytokine was performed by MILLIPLEX^®^ Analyst 5.1 software. A calibration curve for each cytokine was generated by adjusting the Median Fluorescent Intensity (MFI) data obtained for each standard to a 4-parameter logistic equation. The concentrations of each cytokine in the samples were interpolated in the corresponding calibration curve. Besides the pre-established range of the calibration curve, a Detectable Concentration (DC) range was automatically calculated by the analysis software for each curve in each run. This DC measures the true limits of detection (MinDC, MaxDC) for an assay by mathematically determining the empirical limits if an infinite number of standard concentrations were run for the assay under the same conditions, and it provides information about the true range of detection for a specific run.

The concentration of IL-10 was derived from a standard curve (four-parameter logistic curve, logC-OD), which was generated from optical density data and analyzed using GraphPad Prism^®^ 9.0.0 version software. The quantification range of the calibration curve for IL-10 was 0.205–50 ng/mL.

### 2.10. Rectal Swab Collection and Preparation

Rectal swabs were collected from each kitten at 4, 8, 12, 16, 20, 28 and 52 weeks using plastic swabs (Deltalab, Barcelona, Spain, ref. 300252) and immediately stored at −80 °C. Swabs were then shipped on dry ice to Eurofins (NGS Lab Constance; Eurofins Genomics Europe Sequencing GmbH, Constance, Germany) for DNA extraction and sequencing.

### 2.11. Microbiota Analysis

Feces samples from the rectum were used to analyze microbiota. Briefly, after DNA extraction, PCR amplification of the V3-V4 region of the 16S rRNA gene and library preparation were performed with the following primers (with Illumina overhand adapters), forward (5′-GAGAGTTTGATYMTGGCTCAG-3′) and reverse (5′-ACCGCGGCTGCTGGCAC-3′). After purification, sequencing was performed on a MiSeq Personal Sequencer (Illumina, San Diego, CA, USA), 2 × 300 bp paired end reads.

As a first step of sequencing data analysis, reads with ambiguous bases (“N”) were removed. Chimeric reads were identified and removed based on the de novo algorithm of UCHIME as implemented in the VSEARCH package [25,26]. The remaining set of high-quality reads was processed using Minimum Entropy Decomposition (MED) [27,28]. MED provides a computationally efficient means to partition marker gene datasets into Operational Taxonomic Units (OTUs). Each OTU represents a distinct cluster with significant sequence divergence to any other cluster. By employing Shannon entropy, MED uses only the information-rich nucleotide positions across reads and iteratively partitions large datasets while omitting stochastic variation. The MED procedure outperforms classical, identity-based clustering algorithms.

Sequences can be partitioned based on relevant single nucleotide differences without being susceptible to random sequencing errors. This allows a decomposition of sequence datasets with a single nucleotide resolution. Furthermore, the MED procedure identifies and filters random “noise” in the dataset, i.e., sequences with a very low abundance of < 0.02% of the average sample size.

To assign taxonomic information to each OTU, DC-MEGABLAST alignments of cluster representative sequences to the sequence database were performed [29] (Reference database: NCBI_nt (Release 2 August 2019)). A most specific taxonomic assignment for each OTU was then transferred from the set of best-matching reference sequences (lowest common taxonomic unit of all best hits). Hereby, a sequence identity of 70% across at least 80% of the representative sequence was a minimal requirement for considering reference sequences.

### 2.12. Statistical Analysis

A minimum sample size per group of 23 kittens was calculated and required an effect size of 1.25 for reaching the targeted test power. This was based on and adapted from the study of Romano et al., 2007 [8], where the authors investigated the effect of dietary nucleotides on puppies’ immune systems. The final total sample size was set at 50, with 25 kittens per group.

Body measurements, standard hematology analysis, serum antibodies and measurements of cytokine expression were analyzed using Linear Mixed Models with appropriate fixed effect structure and animal as random term.

Diet groups, Time, Gender and their respective interactions were used as fixed effects for the analysis of body measurements. The impact of Diet groups, Time, Condition and their respective interactions was studied for measurements of cytokine expression. Diet groups, Time and their respective interactions were used as fixed effects for the analysis of standard hematology and serum antibodies.

For statistical analysis of the cytokine levels in the different conditions, we first set to 0 all the values that were below the limit of quantification (LoQ). Then, the value corresponding to the unstimulated condition was subtracted from the value of the stimulated condition (either PHA or RA). LoQ and limit of detection (LoD) values are available in Supplementary Table S3.

Tukey HSD was applied for *p*-value correction for multiple comparisons. Significance level was set at 5% after correction for two-sided test. Parameter measurements were Log or rank transformed as appropriate for meeting classical statistical assumptions of the model (normally distributed residuals and homoscedasticity).

Statistical analyses were carried out in R version 4.5.1 [30]. dplyr package was used for data manipulation and ggplot2 for data visualization [31,32]. Linear Mixed Models were calculated using lmer function from lme4 package and emmeans package was used to calculate marginal means and pairwise contrasts [33,34].

Relative abundance graphs were produced using genus taxonomic level table by normalizing the raw counts with the total number of counts among each sample. Alpha diversity was assessed using observed richness (number of OTUs) and the Shannon diversity index, calculated with the vegan package in R. Statistical comparisons between groups (diet, timepoints) were performed using *t*-tests (paired when comparing time series; otherwise, unpaired for diet comparisons) with Bonferroni correction for multiple testing. Beta diversity was evaluated using Bray–Curtis dissimilarity on relative abundance data. Principal Coordinate Analysis (PCoA) was conducted to visualize community structure. Group differences were tested using PERMANOVA (adonis/capscale functions, vegan, 999 permutations), accounting for individual when comparing within diet time series, and group factors when appropriate. Homogeneity of multivariate dispersion among groups (e.g., diet × time) was assessed with the ‘vegan::betadisper’ procedure [35]. An ANOVA on these centroid distances and a permutation test (999 permutations) evaluated global differences in dispersion. When the overall test was significant, pairwise group comparisons of dispersion were obtained via permutation (‘permutest(…, pairwise = TRUE)’). Resulting raw *p*-values from pairwise contrasts were adjusted for multiple testing using the Bonferroni method, controlling the family-wise error rate.

Differential abundance of taxa across groups was analyzed using the DESeq2 package [36]. Negative binomial generalized linear models were fitted, including relevant factors (diet, time, individual), and contrasts of interest were extracted. We added a pseudocount of 1 (data + 1) solely to avoid all-zero rows. Normalized counts shown in figures and exported tables correspond to DESeq2’s internal size-factor normalization (median-of-ratios method; we did not override or supply custom size factors). We relied on the default DESeq2 workflow (DESeq() followed by results()), so independent filtering was applied automatically (independentFiltering = TRUE by default). Multiple testing correction used Bonferroni adjustment (pAdjustMethod = “bonferroni”). Taxa with adjusted *p*-value < 0.05 were considered significant.

## 3. Results

No clinically relevant adverse events were recorded, with the exception of two cases of dermatitis detected in control group cats. Complete datasets were obtained from 49 of 50 kittens. One female kitten from the supplemented group woke up suddenly after neutering, causing an injury in the trachea. The kitten was treated and remained in the study, but no further sampling was performed on this individual from week 28.

### 3.1. Dietary Acceptance, Body Weight and Body Measurements

Acceptance of both diets was good throughout the study. As kittens were group-fed, recording of individual dietary intake was not possible. Linear Mixed Model (LMM) analysis did not show any significant effect for the interaction group–week–gender or gender–group, but showed significant effects for group–week, week–gender and week on mean body weight, thoracic circumference or front leg length (Table 2).

Kittens in both groups gained body weight at expected rates, according to the study of Salt et al., 2023 [37], with no significant differences in body weight gain between dietary treatments.

**Figure 1:**
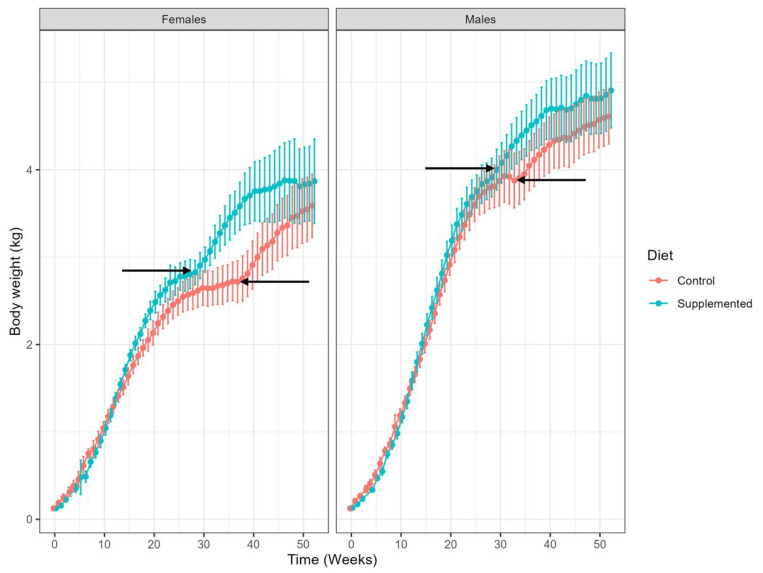
Changes in mean body weight (kg) in females and males from birth (week 0) to 52 weeks of age, showing control (red) and supplemented kittens (blue). Values shown are group means and ranges. Arrows indicate timing of neutering for each group, that are correlated with increased growth trajectories.

There was no significant difference in initial mean body weight between treatment groups for both females and males (Figure 1). At week 4, mean body weight did not differ significantly between treatment groups for females: 0.38 ± 0.09 kg (control) and 0.36 ± 0.07 kg (supplemented; *p* = 0.3848), whereas for males, there was a statistically significant difference between groups: 0.41 ± 0.08 kg (control) versus 0.34 ± 0.04 kg (supplemented; *p* = 0.0003). This is a chance occurrence post-randomization, and the subsequent analysis did not reveal any significant differences in overall growth trajectories between groups, as presented in Figure 1. By week 52, there was no significant effect of treatment group on mean body weight in females or males. Growth trajectories were seen at slightly different timepoints in males and females in control and supplemented groups (Figure 1).

No significant effect of treatment group was found relating to changes in mean thoracic circumference (Supplementary Figure S1) or mean front leg length (Supplementary Figure S2).

### 3.2. Hematology Analysis

Significant differences in certain hematology parameters were found at multiple timepoints between dietary groups (*p* < 0.05). Total erythrocyte counts, hemoglobin and MCHC were significantly increased in the treatment group at several timepoints. The treatment also significantly decreased MCV at various timepoints (Supplementary Table S1). There were no consistently significant effects of the treatment on any other hematology parameters (Supplementary Table S1).

### 3.3. Serum Antibody Responses to Vaccinations

**Figure 2:**
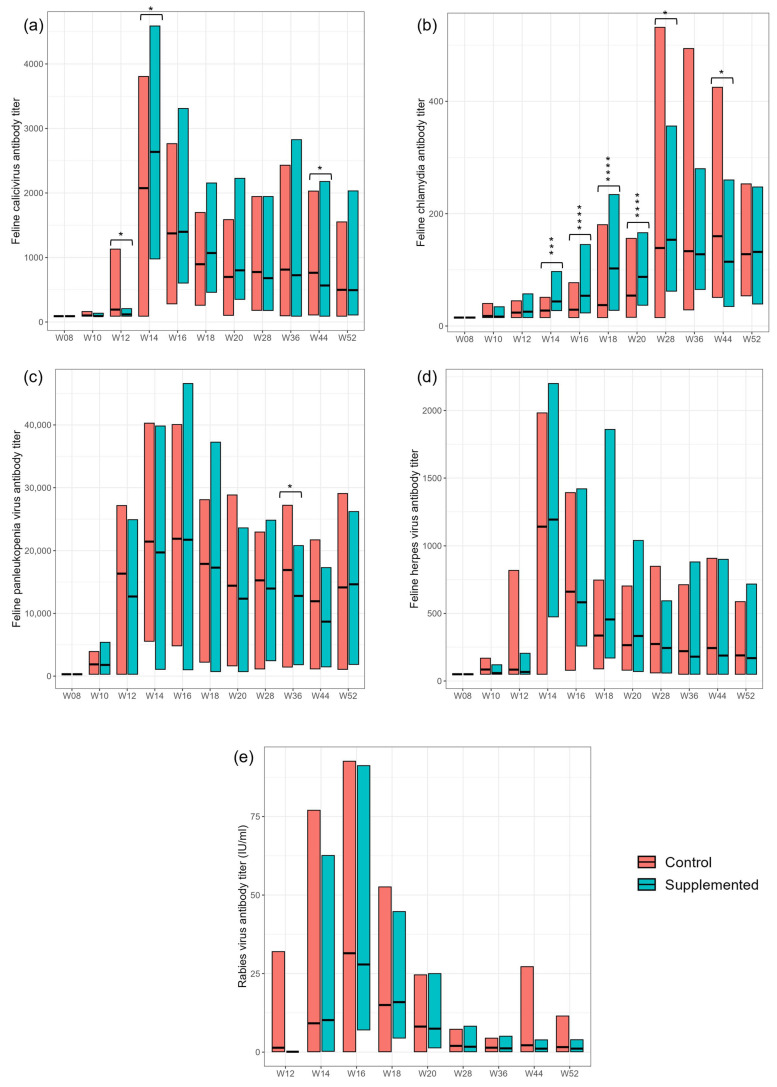
Antibody titers (units) in serum samples collected at weeks 8, 10, 12, 14, 16, 20, 28, 36, 44 and 52 against (**a**) feline calicivirus (FCV); (**b**) feline *Chlamydia*; (**c**) feline panleukopenia virus (FPV); (**d**) feline herpes virus (FHV) and (**e**) rabies virus. Values are presented for control and supplemented dietary groups as means and ranges. Values were significantly different between control and supplemented where * *p* < 0.05; *** *p* < 0.001; **** *p* < 0.0001.

The treatment group showed significantly increased mean antibody response to the *Chlamydia* vaccine at several timepoints from weeks 14 to 44 compared with controls (Figure 2; Supplementary Table S2). However, there was no significant difference between groups by week 52. Following vaccination, antibody titers to FCV, FHV, FPV and rabies were increased in both supplemented and control kittens. However, there was no consistent significant effect of dietary treatment on these responses (Figure 2; Supplementary Table S2).

### 3.4. Serum Cytokines

**Figure 3:**
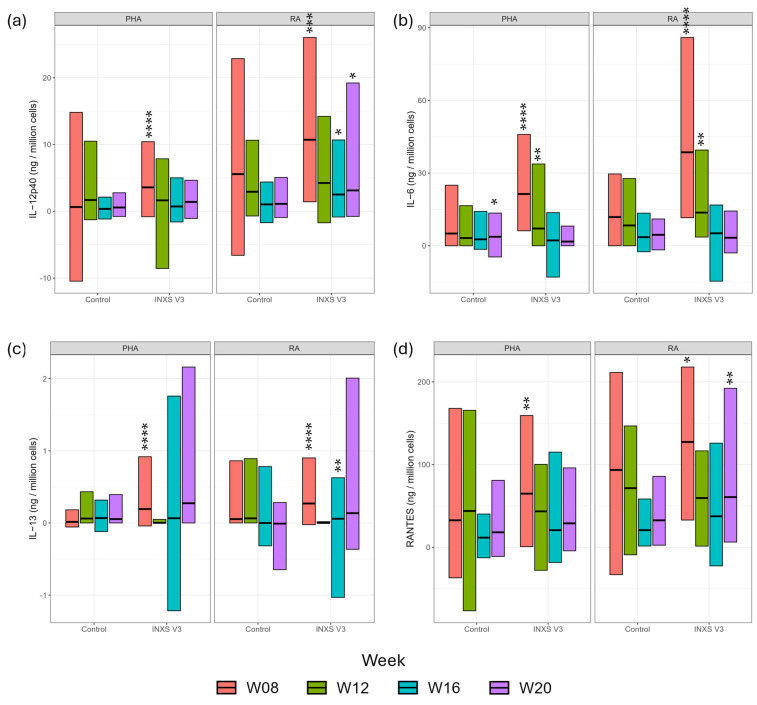
Serum cytokine analysis at week 8 (red), week 12 (green), week 16 (blue) and week 20 (purple) in response to PHA stimulation (positive control) and RA stimulation in control and treatment groups. Data are presented as group means and ranges (**a**) IL-12p40; (**b**) IL-6; (**c**) IL-13; (**d**) RANTES. Mean values for treatment group were significantly different from control group where * *p* < 0.05; ** *p* < 0.01; *** *p* < 0.001; **** *p* < 0.0001.

IL-12p40 (Interleukin 12p40), IL-13 (Interleukin 13), IL-6 (Interleukin 6) and RANTES (Regulated on Activation, Normal T cell Expressed and Secreted) showed strong responses to rabies antigen (RA) stimulation in the treatment group, compared with control (Figure 3 and Supplementary Table S4). There was a weaker response of IL-4 (Interleukin 4), IL-8 (Interleukin 8), KC (Keratinocytes-derived chemokine), MCP-1 (Monocyte Chemoattractant Protein 1) and PDGF-BB (Platelet-Derived Growth Factor BB) at selected timepoints (Supplementary Figure S3 and Table S4) and no effect of treatment on IL-10 (Interleukin 10), IL-18 (Interleukin 18), IL-1b (Interleukin 1 beta), IL-2 (Interleukin 2), FLT-3L (FMS-like tyrosine kinase 3 ligand), GM-CSF (Granulocyte-macrophage colony-stimulating factor) and TNF-a (Tumor Necrosis Factor alpha) (Supplementary Figure S4 and Table S4).

We were unable to perform statistical analysis for four cytokines because of the scarcity of values above the LoQ for most of all timepoints and conditions assessed. Those cytokines were sFas (soluble Fas), IFN-γ (Interferon gamma), SCF (Stem Cell Factor) and SDF-1 (Stromal Cell-Derived Factor 1).

### 3.5. Microbiota Analysis

#### 3.5.1. Relative Abundance

**Figure 4:**
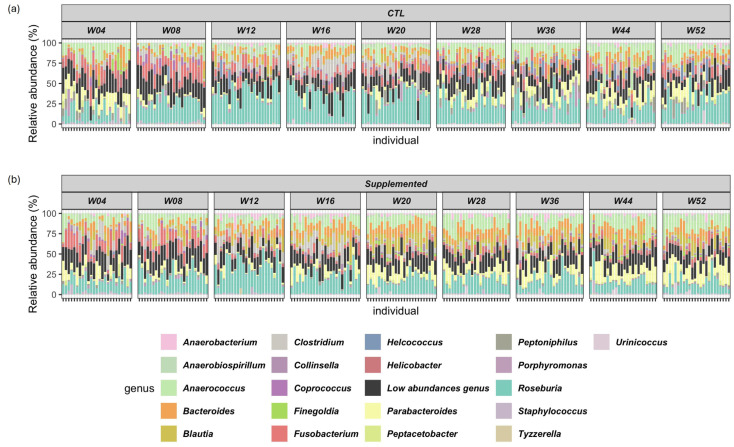
Relative abundance of top twenty genera in control and supplemented kittens from weeks 4 to 52. “Low abundances genus” in black totalize the “minor genera” to have 100% relative abundance. Relative abundance is calculated using the total number of annotated counts at the genus level. (**a**) Control (CTL) group; (**b**) supplemented group.

Relative abundance of the top twenty genera detected in the samples is shown in Figure 4. Predominant fecal bacterial phyla in all cats were *Firmicutes* (control: 56–69%; supplemented: 55–72%), *Bacteroidetes* (control: 11–23%; supplemented: 14–27%), *Proteobacteria* (control: 7–13%; supplemented: 8–10%), *Fusobacteria* (control: 4–12%; supplemented: 2–11%) and *Actinobacteria* (control: 1–6%; supplemented: 1–5%) (Supplementary Table S5).

#### 3.5.2. Alpha Diversity

**Figure 5:**
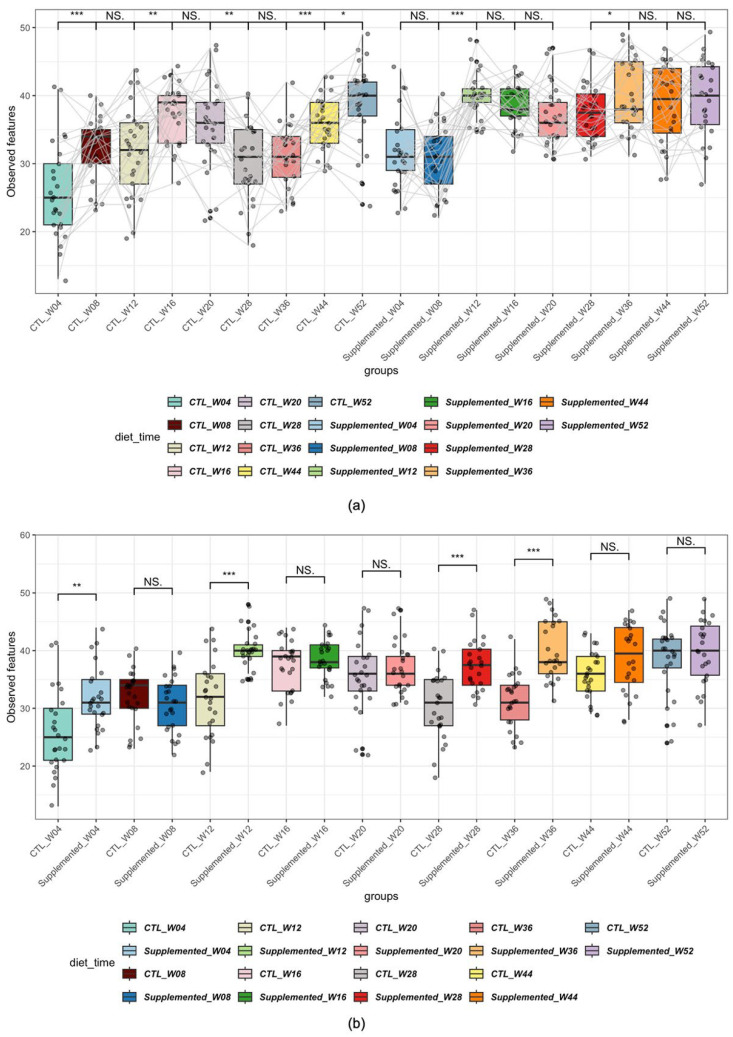
Alpha diversity of samples across diet and time, metrics: Richness. (* *p* < 0.05, ** *p* < 0.01, *** *p* < 0.001; NS: non-significant). (**a**) Comparison by time (the gray lines connect each individual at each timepoint); (**b**) comparison by diet.

Figure 5 shows alpha diversity of samples, comparing observed richness by time (Figure 5a) or by diet (Figure 5b).

Significant increases in richness (i.e., presence/absence of genera) were observed in the control group at week 8 compared to week 4, week 16 compared to week 12, week 44 compared to week 36 and week 44 compared to week 52. A decrease in richness was also evident at week 28 compared to week 20 (Figure 5a). Fewer significant changes were noted over time in the control group for Shannon diversity index, suggesting that the differences observed in richness are due to minor genera of the microbiome (Supplementary Figure S5).

The supplemented group showed significant increases in richness at week 12 compared to week 8 and at week 36 compared to week 28 (Figure 5a). In comparison, Shannon diversity index showed higher diversity in this group at week 16 compared to week 12 and week 20 compared to week 16 (Supplementary Figure S5). The paired analysis between weeks 20 and 28 could not be performed because, as indicated earlier, sampling was stopped on one animal in this group, which prevented evaluation of individual effects. Subsequent comparisons (e.g., week 28 versus week 36 and beyond) excluded this animal.

When compared by dietary group, the supplemented group showed significantly increased microbial richness compared with controls at weeks 4, 12, 28 and 36 (Figure 5b). It is noteworthy to mention that although there was already a significant difference in richness at week 4, this difference is no longer significant at week 8. When comparing using the Shannon diversity index, the supplemented group showed significantly increased alpha diversity at weeks 4, 12, 16, 20, 28, 36 and 52 (Supplementary Figure S6).

#### 3.5.3. Beta Diversity

A PERMANOVA (Permutational Multivariate Analysis of Variance) was conducted to compare the beta diversity between the two groups across the entire experiment. The analysis revealed a statistically significant difference between groups, with the model explaining 21% of the observed variation in beta diversity (R^2^ = 0.21, Table 3).

##### Control Group Time Series Comparison

**Figure 6:**
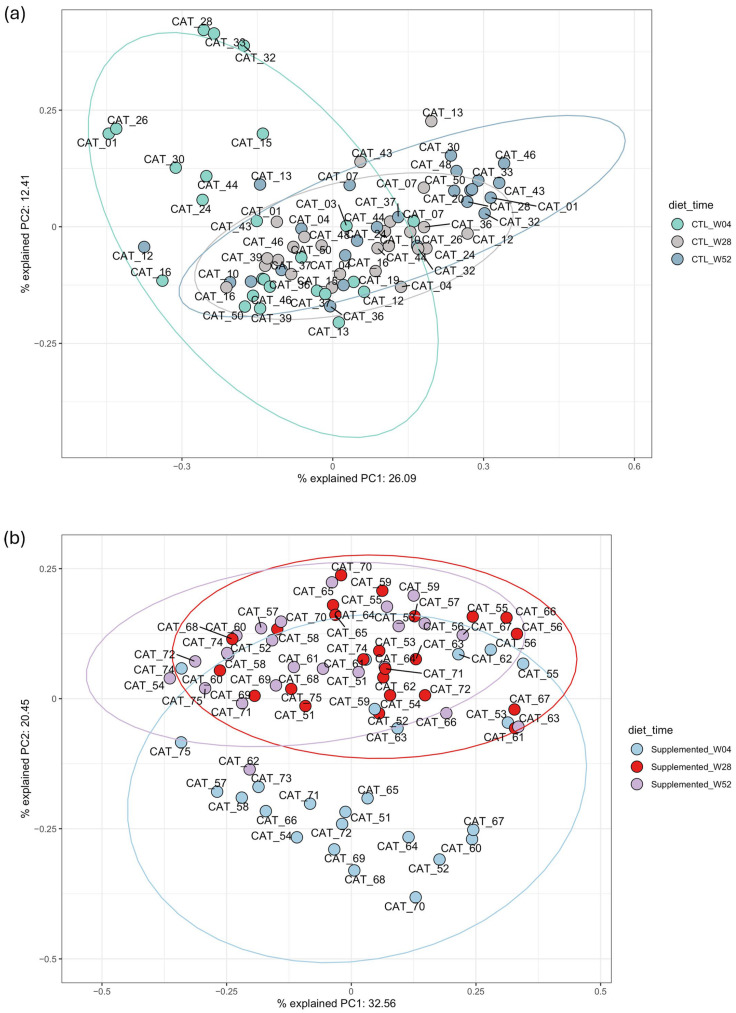
Principal component analysis (PCoA) using weighted beta-Bray–Curtis beta diversity distance. (**a**) Samples from weeks 4, 28 and 52 from control group; (**b**) samples from weeks 4, 28 and 52 from supplemented group. Closeness of points indicates microbiome similarity. Points are color-coded according to timepoint.

PCoA of control group samples demonstrates a similar pattern between clusters of samples from weeks 28 and 52 compared to samples from week 4 (Figure 6a). The PERMANOVA comparisons were significant for almost all the 2 by 2 timepoint comparisons except the comparison between week 44 and week 52 (Supplementary Table S6). The dispersion was not significant between the majority of the comparisons of interest, only the comparison between the control group at week 4 and week 8 were considered significant.

##### Supplemented Group Time Series Comparison

As observed for the control group, PCoA of supplemented group samples demonstrates a similar pattern between clusters of samples from weeks 28 and 52 compared to samples from week 4 (Figure 6b). PERMANOVA comparisons were significant for most of the 2 by 2 timepoints comparisons except week 28 versus week 36 and week 44 versus 52 (Supplementary Table S6). The PERMANOVA results demonstrated a significant difference between the groups when comparing each timepoint two by two, emphasizing an influence of the supplemented diet on the microbiome.

#### 3.5.4. Univariate Analysis

**Figure 7:**
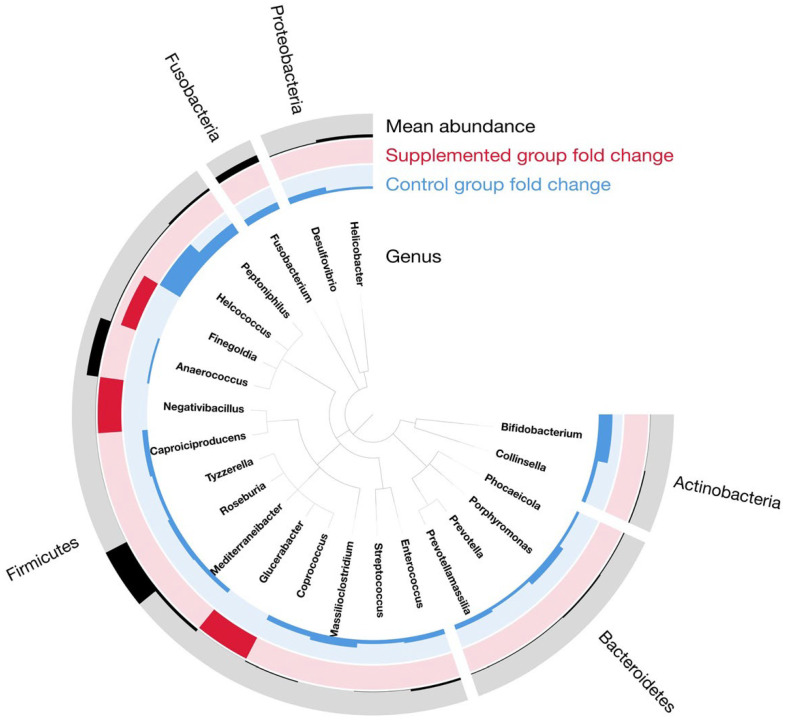
Cladogram representing the phylogeny of the genus discriminating the groups in time series analysis. Black histograms represent the mean abundance of each genus; red and blue histograms represent fold-change value from the DESeq2 analysis where red values are enriched in the supplemented group and blue values are enriched in the control group.

To identify the genera that are different among groups in this time series analysis, the DESeq2 univariate model was applied on the raw genus taxonomic table. To robustly assess the diet effect, the DESeq2 model included “kitten_ID” as a blocking factor to account for repeated measures from the same subject across time, and “time” as a fixed effect. This model structure allows us to test for differences due to diet while controlling for both inter-kitten variation and temporal changes common to all groups. All the relevant genera selected have a corrected *p*-value < 0.05 (Figure 7). In the supplemented group, the three enriched genera corresponded to *Finegoldia*, *Negativibacillus* and *Glucerabacter*. From the twenty depleted bacteria in the supplemented group, *Helcococcus* was the most discriminant with a negative log2 fold-change close to 5. *Peptoniphilus* and *Bifidobacterium* also have a noteworthy negative log2 fold-change superior to two. All other significant genera had minor changes between the diets, but this can be due to minor abundance in the microbiome. The cladogram representing these findings is shown in Figure 7.

Most of the impacted genera belonged to the *Firmicutes* phylum, but the differential analysis also recovered some genera related to *Bacteroidetes* (four genera), *Actinobacteria* (two genera), *Fusobacteria* (one genus) and *Proteobacteria* (two genera). The complete description of the statistics resulting from the DESeq2 model application is presented in Table 4.

## 4. Discussion

This study aimed to test the effect of a diet supplemented with nucleotides, scFOS, XOS, β-carotene and vitamin E on immune function and the gut microbiota in kittens from beginning of weaning at 4 weeks of age up to 52 weeks of age.

We have previously shown in a study of kittens up to 28 weeks that the dietary supplement as tested here enhances antibody-mediated response to vaccination, suggesting a role for this diet in immune support [21]. The current study extends these findings to kittens 52 weeks of age and additionally demonstrates an effect of the supplement on several cytokines and on the gut microbiota.

Body weight and body measurements monitored throughout the study showed that kittens grew normally with no significant differences between supplemented and control groups. Together with lack of reported adverse effects in association with the test diet, these findings suggest that the dietary supplement has no detrimental effects on growing kittens. The earlier neutering times in the supplemented group (28 weeks for females and 26 weeks for males) coincided with an increase in weight gain at these timepoints; this growth spurt was seen later in the control group in line with the later neutering times of 39 weeks (females) and 32 weeks (males). This growth trajectory in kittens following neutering has been reported previously and has been observed to be more dramatic in females compared with males [37,38].

Although the supplement resulted in significantly increased total erythrocyte count, hemoglobin and MCHC, overall, the values stayed within normal reference ranges [39,40]. Kittens often show progressive increases in erythrocyte count and hemoglobin as they mature. This is indicative of normal adaptations to higher metabolic demand as kittens grow and become more active. The increase in MCHC within normal limits could reflect maturation of red cells and improved hemoglobin packing. Taken together, those data suggest that the red blood cells of supplemented kittens could be more efficient at carrying oxygen. There were no consistent effects of the supplement on lymphocytes, although dietary nucleotides have been reported to influence lymphocyte proliferation [41].

Vaccine responses can be used as clinically relevant biomarkers of the immunological response to challenge and can be interpreted as a surrogate marker of a typical immune response to infection. Kittens in both dietary groups mounted an antibody response to all five vaccines with the exception of one female (kitten 12, control group) that was either omitted on the day of rabies vaccination or did not mount an antibody response against the rabies vaccine. A significant difference in response as a result of supplementation was only observed for the *Chlamydia* vaccine such that a greater and more sustained antibody response was observed. Supplemented kittens mounted a faster antibody response to the *Chlamydia* vaccine and demonstrated significantly improved mean serum antibody levels at weeks 14, 16, 18, 20, 28 and 44 versus controls. Increased corresponding antibody levels following vaccination are associated with improved resistance against infection. There is no known protective antibody titer for *Chlamydia* as it is not a routine vaccination, so it is not possible to comment on whether the supplement improved protection against this bacterium. The vaccination reduces clinical signs of infection, thereby reducing risk of transmission and may be beneficial in multi-cat households and shelters. When comparing our study with similar studies, including that of Atwal et al., 2023 [21], which used the same supplementation cocktail, the strains and types of vaccines used and the methods employed to monitor vaccine responses are different, hampering direct comparison of the data. However, our findings are generally consistent with three similar studies that demonstrate a beneficial effect of dietary nucleotides on responses of cats and dogs to vaccination [8,21,42].

Although there was no consistent effect of supplementation on antibody responses to the rabies, FCV, FHV and FPV vaccinations, supplemented kittens showed significantly improved levels of several serum cytokines in response to RA. This suggests an enhanced cellular response to RA in this group, despite a lack of distinction between supplemented and control groups in terms of rabies antibody response to the corresponding vaccine. Some of the key cytokines influenced by the supplement in the whole blood assay were linked to a bacterial immune response (IL-12p40 and IL-6); this concurs with the boosted antibody titer to the *Chlamydia* vaccination, the only bacterial vaccine used in the study. However, expression of the same cytokines was also triggered by RA stimulation. The reasons for this are unclear but this is a potentially interesting observation which may stimulate further research. These findings indicate that the conventional use of antibody titer as a measure of vaccination effectiveness may be insufficient in certain circumstances and additional markers of cellular immunity such as cytokine production should be considered.

The supplementation had particularly notable effects on IL-12p40, IL-13, IL-6 and RANTES expression levels after stimulation with RA. IL-12p40 (natural killer cell stimulatory factor 2) is a structural component of two key cytokines, IL-12 and IL-23 [43]. IL-12 drives T-helper type 1 (Th1) responses, essential for the development of cell-mediated immunity against intracellular pathogens such as viruses. IL-23 plays a critical role in maintaining and expanding Th17 cells, which are involved in immunity against bacteria and fungi. When a kitten’s immune system responds to a virus, both IL-12 and IL-23 are released, both of which require IL-12p40 for their production. The IL-12 family have a key involvement in ensuring the appropriate immune response to vaccination. There were significant increases in IL-12p40 response to RA in the supplemented group at weeks 8, 16 and 20 compared with controls. Weeks 16 and 20 corresponded with highly significant increased *Chlamydia* antibody titers in the supplemented group at these timepoints.

IL-13 is a cytokine mainly produced by activated T-helper type 2 (Th2) cells [44]. This cytokine has structural and functional similarities with IL-4 [45]. IL-13 is known to inhibit the production of pro-inflammatory cytokines and to induce the proliferation of macrophages to the M2 phenotype, which helps modulate Th2 immune response [46], which is also modulated in the supplemented group after RA stimulation. In our study, IL-13 levels were significantly higher after RA stimulation in treatment versus control kittens at weeks 8 and 16, suggesting an effect of the supplement on the resolution of inflammation post-stimulation. IL-6 is released in response to infection and is a potent inducer of acute phase response protein production in the liver. IL-6 is an important mediator of fever, a critical infection defense mechanism in young animals [47]. Known to have a central role in activation and regulation of the innate and adaptive immune system, IL-6 has duel pro- and anti-inflammatory effects, dependent on the pathway activated. The significantly elevated levels of IL-6 after RA stimulation in the treatment group compared with control at weeks 8 and 12 suggest improved activation of the immune system. RANTES is a chemoattractant for T cells, eosinophils and basophils, playing an active role in recruiting leukocytes into inflammatory sites. RANTES mRNA expression was shown to increase in the skin of cats with eosinophilic plaques compared with non-affected cats, demonstrating the key role of RANTES in eosinophil infiltration in these animals [48]. In the current study, RANTES levels were significantly higher after RA stimulation in treatment versus control kittens at weeks 8 and 20, indicating an effect of the supplement on sustaining the inflammatory response.

Comparisons of alpha diversity in gut microbiota across treatments were analyzed using richness and Shannon diversity indices. A general increase in microbial diversity was seen over time in both groups of kittens, an observation consistent with those made in kittens and babies during early growth [49]. Alpha diversity was already higher for the supplemented group versus control at week 4, which impaired observation of a richness gain at the beginning of the treatment period. Whilst richness was significantly increased in the supplemented group compared with controls at four of the nine analyzed timepoints, Shannon diversity index showed significant increases in alpha diversity in the supplemented group at seven of the nine timepoints. The supplemented kittens also showed significantly higher Shannon diversity at the end of the study. Overall, the supplement tested here elicited a positive effect on alpha diversity of the gut microbiota. A diverse gut microbiota promotes a broad array of enzymes and biochemical pathways that would otherwise not exist in the host, increasing availability of energy and nutrients [50]. In addition, a varied gut microbial population has been associated with reduced relative abundance of pathogens in free-roaming cats [51].

The predominant phyla found in the gut microbiota of kittens in the current study were *Firmicutes*, *Bacteroidetes*, *Proteobacteria*, *Fusobacteria* and *Actinobacteria*, in agreement with those reported in the literature [52,53,54]. The relative proportions of these phyla can vary by individual and are dependent on several factors including diet, age, living environment and analysis techniques used [53,55].

Beta diversity analysis suggested that the microbial composition evolved very quickly; univariate analysis showed a total of twenty depleted genera and three enriched genera, with statistically significant differences between dietary groups. In the supplemented kittens, relative abundances of *Finegoldia*, *Negativibacillus* and *Glucerabacter* were increased. The *Finegoldia* genus has been previously described as one of the most predominant genera in the gut microbiota of healthy young cats [56]. The *Negativibacillus* genus was first characterized from a human colon isolate and has since been reported as one of 30 core genera in the gut microbiota of healthy cats [53,57]. *Glucerabacter* have been isolated from dog feces and are suggested to play a major role in the hydrolysis of glucosylceramide in the canine intestine [58].

Twenty genera were depleted in the supplemented group compared to controls. The three genera showing the most notable depletions in the supplemented group compared to control were *Helcococcus*, *Peptoniphilus* and *Bifidobacterium*. The *Helcococcus* genus is described as an opportunistic pathogen in different animals [59]. *Peptoniphilus* has been referenced as an opportunistic pathogen in humans [60]; in cats, this genus has been detected in moderate relative abundance in the cat anal gland microbiota [61]. In this context, a reduction in the relative abundance of putative pathobionts such as *Helcococcus* and *Peptoniphilus* may reflect a shift toward a less proteolytic or less inflammation prone community, although causality cannot be inferred from the present data. XOS and FOS are prebiotic substrates for saccharolytic fermentation that produce SCFAs (Short Chain Fatty Acids) such as lactate and acetate, which can lead to light acidification of the environment. This could be one explanation for *Helcococcus* and *Peptoniphilus* decrease in the gut microbiota of supplemented kittens. Additional measures, such as fecal pH or SCFAs quantification, would be required to validate this hypothesis.

The *Bifidobacterium* genus has been suggested as one of the key probiotics involved in maintaining the health of kittens following weaning [54]. The relative abundance of this genus appeared to be reduced as a result of supplementation, but the interpretation of this decrease needs to be nuanced. Lower relative abundance does not necessarily imply diminished ecological function or adverse health outcomes. First, relative abundance is influenced by expansions of other fermentative taxa; a proportional decrease can occur even when absolute counts are stable. Second, fiber type, dose and matrix can differentially select for saccharolytic communities in carnivores, with non-*Bifidobacterium* taxa (e.g., certain *Lactobacillus* or *Clostridiales* members) capable of fermenting oligosaccharides and participating in cross feeding networks that yield similar end products (e.g., acetate, lactate and downstream butyrate). Third, *Bifidobacterium* composition is strain-specific and age-dependent; natural post-weaning trajectories often involve shifts in Bifidobacterial representation as dietary protein and fat increase, without necessarily indicating a decline in gut health. In our study, the *Bifidobacterium* genus remained detectable across groups, and the observed reduction may reflect temporal fluctuation and competitive niche adjustment in response to the supplemented diet rather than a deleterious effect per se.

The supplement tested in the current study comprises a cocktail of ingredients; therefore, it is not possible to attribute the observed effects to a single ingredient. It is also not possible from this study to determine whether any of the ingredients act synergistically, and further work would be needed to better understand the contribution of the different components of the cocktail on all the parameters assessed in this study. However, some of the observed effects of the supplement are in agreement with the literature when considering the activity of individual components.

The developing kitten is especially vulnerable to diarrhea and other gastrointestinal disturbances which can be triggered by stress, dietary changes and infections. Dietary input can play a key role in influencing gut microbial composition in growing animals and the observed effects on the microbiota are likely due to the prebiotic effects of scFOS and XOS contained in the supplement. Previous studies have demonstrated the beneficial effects of FOS and XOS on gut bacterial populations in cats [10,62,63,64]. The gut microbiota is known to influence both the development and functioning of the immune system in cats, and disruption of the gut microbiota (dysbiosis) disturbs immune homeostasis [65]. It is therefore possible that some of the changes in the gut microbiota elicited by the scFOS and XOS content of the supplement may have been partly responsible for some of the effects observed on immune parameters.

Vitamin E is a powerful antioxidant and has been shown to benefit immune function in cats when supplemented to the diet in excess of essential requirements [18]. It is thought to achieve this by combatting free radicals and lipid peroxidation, which are known to suppress the immune system [66]. The β-carotene component of the supplement is also likely to have contributed an antioxidant effect and has been described as having a role in regulating both cell-mediated and humoral immune responses in animals and humans [20,67].

The main strength of this study is the randomized, controlled, double-blinded design which minimizes bias and increases confidence in the data. This is further supported by the use of multiple diversity metrics in the microbiota analysis including the highly sensitive Bray–Curtis metric to allow differentiation between dietary groups. However, it is also acknowledged that the study has some limitations. The population size may not have been sufficient to observe the expected effects of the treatment, considering the high inter-individual variation seen in terms of antibody responses and hematological measures. The kittens used in this study were SPF and were therefore not exposed to the range of viruses and bacteria that would typically be encountered by conventionally housed kittens. This limited exposure to pathogens impairs immune training and means that SPF kittens may have a different immunological response to antigens compared with pet kittens. Testing the supplement in privately owned kittens would allow better representation of typical environmental conditions of pet kittens, known to influence immunity and gut microbiota. The two groups of kittens were neutered at different times due to constraints linked to the facility. Neutering has an impact on metabolism and could have affected the outcome of this study [37,38]. To date, no clear link has been shown between neutering and response to vaccine, and there may be a link between neutering and microbiota shifts in cats [68,69]. Further research is needed to elucidate those hypotheses. The approach of using rectal swabs to examine the gut microbiota was chosen for practicality but may have affected the results. Fecal samples are considered the gold standard method for analyzing gut microbiota but require collection of fresh samples for optimal results. Rectal swabs capture smaller samples sizes of feces and therefore can result in lower DNA yield and limit taxon detection. However, a comparison made between the two methods indicated no significant differences in key alpha and beta diversity measures or in abundance of major annotated phyla, suggesting rectal swabs are a suitable method for compositional and functional analyses of gut microbiota [70]. The study should be repeated in client-owned kittens to confirm applicability to the pet population.

## 5. Conclusions

A dietary supplement containing nucleotides, scFOS, XOS, β-carotene and vitamin E enhanced response to *Chlamydia* vaccination in kittens, improved several cytokine responses and promoted changes to the gut microbiota with no associated adverse effects. Based on these findings, together with those of a previous study testing the same supplement, this cocktail of ingredients could be a beneficial addition to kitten diets. The study findings also suggest that further research is needed to better understand the link between gut microbiota and the response to vaccination.

## Figures and Tables

**Table 1:** Nutrient composition of control and test diets. Dietary analysis was performed at Eurofins Ltd. The values presented in this table are averages and are within conformance standards.

Proximate Analysis (as Fed)	Control Diet (1)	Control Diet (2)	Test Diet
Moisture (%)	5.2	4.8	5.5
Crude protein (%)	32.2	32.4	31.7
Crude fat (%)	18.3	18.1	18.6
Total dietary fiber (%)	8.5	8.1	7.0
Ash (%)	6.5	6.9	6.2
Gross Energy (kcal/g)	5.04	5.05	5.00
Sodium (%)	0.40	0.40	0.41
Phosphorus (%)	0.92	0.93	0.95
Potassium (%)	0.63	0.60	0.61
Magnesium (%)	0.07	0.09	0.09
Sulfur (%)	0.45	0.46	0.38
Calcium (%)	1.04	1.03	1.03
Chloride (%)	0.77	0.75	0.69
Oxalic acid (ppm)	522	492	355

**Table 2:** Linear Mixed Model (LMM) analysis summarizing the results on fixed effects for body weight, front leg length and thoracic circumference.

Fixed Effect	Body Weight			Front Leg Length			Thoracic Circumference		
	Chisq	Df	*p*-Value	Chisq	Df	*p*-Value	Chisq	Df	*p*-Value
(Intercept)	0.99	1	0.3203	1963.02	1	< 0.0001	630.76	1	< 0.0001
Group	0.00	1	0.9802	1.20	1	0.2731	0.04	1	0.8368
Week	7291.46	50	< 0.0001	1410.41	21	< 0.0001	817.94	21	< 0.0001
Gender	0.00	1	0.9943	5.28	1	0.0215	4.91	1	0.0267
Group:week	324.72	50	< 0.0001	124.00	21	< 0.0001	131.07	21	< 0.0001
Group:gender	0.00	1	0.9612	1.99	1	0.1579	1.25	1	0.2637
Week:gender	1106.79	50	< 0.0001	108.08	21	< 0.0001	51.72	21	0.0002
Groupweek:gender	49.59	50	0.4897	18.30	21	0.6301	15.85	21	0.7783

**Table 3:** PERMANOVA results for beta diversity comparison.

	Df	SumOfSqs	R^2^	F	Pr(>F)
Model	9	12.03	0.21	12.66	0.001
Residual	436	46.03	0.79	NA	NA
Total	445	58.06	1.00	NA	NA

Df: Degree of freedom; SumOfSqs: Sum of Squares; R^2^: Coefficient of Determination (R-squared); F: F-statistic; Pr(>F): *p*-value.

**Table 4:** Differential abundance analysis results from DESeq2 model.

Taxa	baseMean	log2FoldChange	lfcSE	stat	*p*-Value	padj
*Helcococcus*	291.246	−4.989	0.305	−16.370	3.15 × 10^−60^	1.42 × 10^−58^
*Peptoniphilus*	744.058	−2.468	0.197	−12.549	4.01 × 10^−36^	1.80 × 10^−34^
*Bifidobacterium*	63.359	−2.897	0.250	−11.596	4.32 × 10^−31^	1.94 × 10^−29^
*Fusobacterium*	2104.226	−1.544	0.138	−11.176	5.37 × 10^−29^	2.42 × 10^−27^
*Roseburia*	6959.130	−1.090	0.102	−10.661	1.55 × 10^−26^	6.95 × 10^−25^
*Massilioclostridium*	6.572	−1.705	0.186	−9.162	5.08 × 10^−20^	2.29 × 10^−18^
*Porphyromonas*	468.569	−1.534	0.168	−9.122	7.37 × 10^−20^	3.32 × 10^−18^
*Desulfovibrio*	279.072	−1.379	0.190	−7.253	4.07 × 10^−13^	1.83 × 10^−11^
*Mediterraneibacter*	877.543	−0.919	0.135	−6.807	9.99 × 10^−12^	4.50 × 10^−10^
*Coprococcus*	286.099	−1.126	0.206	−5.470	4.51 × 10^−8^	2.03 × 10^−6^
*Prevotellamassilia*	221.725	−1.066	0.202	−5.278	1.31 × 10^−7^	5.87 × 10^−6^
*Caproiciproducens*	40.556	−1.133	0.216	−5.244	1.57 × 10^−7^	7.06 × 10^−6^
*Enterococcus*	669.909	−1.107	0.217	−5.104	3.33 × 10^−7^	1.50 × 10^−5^
*Helicobacter*	903.391	−0.530	0.120	−4.414	1.01 × 10^−5^	4.57 × 10^−4^
*Glucerabacter*	26.445	1.051	0.243	4.330	1.49 × 10^−5^	6.70 × 10^−4^
*Phocaeicola*	290.165	−0.602	0.140	−4.303	1.69 × 10^−5^	7.59 × 10^−4^
*Collinsella*	332.341	−0.857	0.202	−4.243	2.20 × 10^−5^	9.91 × 10^−4^
*Negativibacillus*	60.975	1.047	0.260	4.019	5.84 × 10^−5^	0.00263
*Streptococcus*	88.353	−0.830	0.213	−3.904	9.44 × 10^−5^	0.00425
*Prevotella*	228.058	−0.624	0.170	−3.673	2.39 × 10^−4^	0.01077
*Anaerococcus*	3429.321	−0.447	0.135	−3.310	9.34 × 10^−4^	0.04204
*Tyzzerella*	23.522	−0.720	0.219	−3.289	0.001005	0.04524
*Finegoldia*	300.111	0.702	0.215	3.262	0.001105	0.04975

Taxa: genera with significant change; baseMean: average DESeq2 normalized abundance across samples; log2FoldChange: log2-transformed change between conditions; lfcSE: standard error of log2FoldChange; stat: Wald test statistic; *p*-value: nominal significance; padj: *p*-value adjusted by Bonferroni correction.

## Data Availability

The data associated with this article will be shared upon reasonable request to the corresponding author. The data are not publicly available, because the supplement is under development and the subject of a patent application.

## Supplementary Materials

The following supporting information can be downloaded at: https://www.mdpi.com/article/10.3390/ani15233504/s1, Table S1: Hematology values shown as medians and ranges for control and supplemented kitten groups. Table S2: Antibody titers in response to vaccinations shown as medians and ranges for control and supplemented kitten groups. Table S3: Limit of quantification and limit of detection of all the cytokines tested. Table S4: Cytokine expressions from the whole blood assays shown as medians and ranges for control and supplemented kitten groups. Table S5: Relative bacterial phyla composition (%) of control and supplemented groups at all timepoints sampled (weeks 4, 8, 12, 16, 20, 28, 36 and 52). Table S6: PERMANOVA comparisons of beta diversity of all samples. Figure S1: Changes in mean thoracic circumference (cm) in females and males from 12 to 52 weeks of age, showing control (red) and supplemented (blue) kittens. Figure S2: Changes in mean front leg length (cm) in females and males from 12 to 52 weeks of age, showing control (red) and supplemented (blue) kittens. Figure S3: Serum cytokine analysis showing a weaker response to RA stimulation at week 8 (red), week 12 (green), week 16 (blue) and week 20 (purple) in response to PHA stimulation (positive control) and RA stimulation in control and treatment groups. Figure S4: Serum cytokine analysis showing no response to RA stimulation at week 8 (red), week 12 (green), week 16 (blue) and week 20 (purple) in response to PHA stimulation (positive control) and RA stimulation in control and treatment groups. Figure S5: Alpha diversity of samples across diet and time, compared by time, metrics: Shannon index. Figure S6: Alpha diversity of samples across diet and time, compared by diet, metrics: Shannon index. Figure S7: Boxplot of the beta dispersion analysis. Boxplots show the distribution of Bray–Curtis distances from samples to their group centroid (betadisper) for all diet–time categories, summarizing multivariate dispersion (beta diversity variability) across groups. ARRIVE checklist.

## Abbreviations

The following abbreviations are used in this manuscript:

DC	Detectable concentration
FCV	Feline calicivirus
FHV	Feline herpes virus
FPV	Feline panleukopenia virus
GM-CSF	Granulocyte-macrophage colony-stimulating factor
IL	Interleukin
KC	Keratinocyte-derived chemokine
LoD	Limit of Detection
LoQ	Limit of Quantification
MCH	Mean corpuscular hemoglobin
MCHC	Mean corpuscular hemoglobin concentration
MCP-1	Monocyte chemoattractant protein-1
MCV	Mean corpuscular volume
MED	Minimum entropy decomposition
OTU	Operational taxonomic unit
PHA	Phytohemagglutinin
PCoA	Principal coordinate analysis
PDGF-BB	Platelet-derived growth factor-BB
RA	Rabies antigen
RANTES	Regulated on activation, normal T cell expressed and secreted
SCF	Stem cell factor
SCFA	Short Chain Fatty Acid
scFOS	Short chain fructooligosaccharide
SDF-1	Stromal cell-derived factor 1
sFas	Soluble Fas
SPF	Specific pathogen free
TNF-α	Tumor necrosis factor α
XOS	Xylooligosaccharides
