# Supplementary material for: A Dietary Supplement Containing Nucleotides, Oligosaccharides, Vitamin E and β-Carotene Promotes Immune Response and Gut Microbiota Changes in Kittens
Source: Animals (Basel). 2025 Dec 4;15(23):3504. doi: 10.3390/ani15233504 (PMC12691447; doi:10.3390/ani15233504)
Supplement: Supplementary file 1 [file animals-15-03504-s001.zip › animals-3985034-supplementary.pdf]

List of the Supplementary Materials:

<u>Tables:</u>

Supplementary Table S1: Hematology values shown as medians and ranges for control and supplemented kitten groups.

Supplementary Table S2: Antibody titers in response to vaccinations shown as medians and ranges for control and supplemented kitten groups.

Supplementary Table S3: Limit of quantification and limit of detection of all the cytokines tested.

Supplementary Table S4: Cytokine expressions from the whole blood assays shown as medians and ranges for control and supplemented kitten groups.

Supplementary Table S5: Relative bacterial phyla composition (%) of control and supplemented groups at all timepoints sampled (weeks 4, 8, 12, 16, 20, 28, 36 and 52).

Supplementary Table S6: PERMANOVA comparisons of beta diversity of all samples.

<u>Figures:</u>

Supplementary Figure S1: Changes in mean thoracic circumference (cm) in females and males from 12 to 52 weeks of age, showing control (red) and supplemented (blue) kittens.

Supplementary Figure S2: Changes in mean front leg length (cm) in females and males from 12 to 52 weeks of age, showing control (red) and supplemented (blue) kittens.

Supplementary Figure S3: Serum cytokine analysis showing a weaker response to RA stimulation at week 8 (red), week 12 (green), week 16 (blue) and week 20 (purple) in response to PHA stimulation (positive control) and RA stimulation in control and treatment groups.

Supplementary Figure S4: Serum cytokine analysis showing no response to RA stimulation at week 8 (red), week 12 (green), week 16 (blue) and week 20 (purple) in response to PHA stimulation (positive control) and RA stimulation in control and treatment groups.

Supplementary Figure S5: Alpha diversity of samples across diet and time, compared by time, metrics: Shannon index.

Supplementary Figure S6: Alpha diversity of samples across diet and time, compared by diet, metrics: Shannon index.

Supplementary Figure S7: Boxplot of the beta dispersion analysis. Boxplots show the distribution of Bray–Curtis distances from samples to their group

centroid (betadisper) for all diet–time categories, summarizing multivariate dispersion (beta diversity variability) across groups.

Supplementary Table S1. Hematology values shown as medians and ranges for control and supplemented kitten groups. *p*-values represent comparison between dietary groups at the sample timepoint, where values in bold denote statistical significance at $p < 0.05$. Band neutrophil and basophil counts not shown as all values were negligible.

Parameter	Week	Control			Supplemented			*p*-value
		n	Median	Range	n	Median	Range	
Mean Corpuscular Hemoglobin (MCH; pg)	8	25	14.10	13.25 -17.82	25	14.58	13.03 -16.33	0.752
	10	25	13.57	12.07 -15.90	25	13.90	12.08 -16.08	0.226
	12	25	12.79	11.51 -19.78	25	12.97	10.65 -14.38	0.816
	14	25	12.09	10.49 -13.90	25	12.20	10.69 -13.11	0.748
	16	25	11.97	10.85 -13.46	25	11.93	10.32 -13.46	0.599
	18	25	11.72	9.66 -13.42	25	11.78	10.20 -12.94	0.890
	20	25	11.66	10.20 -13.47	25	11.59	10.18 -13.23	0.503
	28	25	11.96	10.52 -14.40	24	11.82	10.29 -13.30	0.276
	36	25	12.20	10.75 -14.65	24	12.53	9.94 -14.53	0.581
	44	25	12.74	10.88 -14.91	24	12.78	11.58 -14.60	0.688
	52	25	12.40	10.08 -14.56	24	12.88	11.56 -15.00	**0.016**
Mean Corpuscular Hemoglobin Concentration (MCHC; g/dl)	8	25	31.90	30.48 - 33.63	25	34.02	30.84 - 36.45	**0.000**
	10				25		30.46 -	**0.016**
		25	31.07	28.30 - 33.75		32.09	34.41	
	12	25	31.80	28.27 - 36.80	25	29.89	28.13 - 35.94	**0.048**
	14	25	32.09	30.17 – 35.00	25	33.33	31.56 - 37.78	**0.000**
	16	25	31.37	28.38 - 33.87	25	36.60	34.33 - 41.08	**0.000**
	18	25	30.70	29.38 - 32.60	25	34.41	32.92 - 41.42	**0.000**
	20	25	32.53	30.54 - 35.05	25	34.97	31.36 - 37.46	**0.000**
	28	25	34.03	31.73 - 36.36	24	36.8	32.95 - 39.53	**0.000**
	36	25	34.36	30.54 - 36.55	24	40.03	36.86 - 45.35	**0.000**
	44	25	38.29	35.42 - 42.65	24	31.94	30.1 - 33.91	**0.000**
	52	25	37.54	33.33 - 42.04	24	33.11	31.35 - 35.71	**0.000**
Mean Corpuscular Volume (MCV; fl)	8	25	44.22	39.97 - 53.45	25	42.88	36.9 - 47.50	**0.062**
	10	25	42.96	38.78 - 48.18	25	42.74	36.91 - 47.69	**0.962**
	12	25	40.66	35.26 - 53.76	25	42.35	31.96 - 46.75	**0.350**
	14	25	38.08	31.85 - 43.45	25	36.22	31.86 - 39.16	**0.011**
	16	25	38.28	32.85 - 44.96	25	32.16	28.34 - 38.89	**0.000**
	18	25	37.64	31.49 - 43.44	25	33.59	28.63 - 38.95	**0.000**
	20	25	35.41	29.1 - 41.23	25	33.48	28.07 - 39.89	**0.001**
	28	25	35.77	30.52 - 41.40	24	32.36	27.21 - 40.37	**0.001**

	36	25	36.19	30.45 - 42.78	24	31.12	24.06 - 35.91	**0.000**
	44	25	33.06	26.72 - 41.50	24	39.86	35.56 - 43.8	**0.000**
	52	25	33.02	27.68 - 41.36	24	39.49	33.3 - 42.94	**0.000**
Eosinophil count (cells/µl)	8	25	734.4	384.5 - 1284	25	1155	760 - 1680	**0.000**
	10	25	569.6	216 - 1464	25	1053.8	392 - 1620	**0.000**
	12	25	762	387 - 3045	25	1090	223.2 - 1812	**0.029**
	14	25	952	692 - 1770	25	1313	730 - 2130	**0.038**
	16	25	1071	546 - 1722	25	1088	605 - 3290	0.741
	18	25	884.7	448 - 1806	25	1080	318 - 2784	0.130
	20	25	896	140 - 2115	25	969.1	470.4 - 2655	**0.043**
	28	25	882.9	398 - 1476	24	1489	665.5 - 4200	**0.000**
	36	25	846	54.2 - 1881	24	1046	630 - 2352	**0.021**
	44	25	806	174.2 - 1980	24	885	278.5 - 2376	0.096
	52	25	600	242 - 1550	24	757	198.9 – 4416	0.107
Lymphocyte count (cells/µl)	8	25	4140	2385.6 - 8607	25	3450	1768.2 - 5904	0.061
	10	25	4264	2538 - 7830	25	4119	2240 - 6612	0.325
	12	25	5289	2595.4 - 11252	25	4290	1936 - 11826	**0.002**
	14	25	5610	2141.3 - 15884	25	3922	2032.8 - 7738	**0.004**
	16	25	5814	3626 - 7396	25	5480	2268 - 12610	0.401
	18	25	4644	2867.2 - 19860	25	4860	2205 - 6882	0.449
	20	25	6380	3358 - 11088	25	4401	2956.3 - 7904	**0.000**
	28	25	4546.8	2534.4 - 9291	24	5901	2117.5 - 8532	0.236
	36	25	3808	1517.6 - 10152	24	5737	2222 - 11280	**0.000**
	44	25	4610.4	1853.8 - 7965	24	4517	1910 - 9900	0.808
	52	25	5032	2606.8 - 7285	24	4041	1232.7 - 6513	**0.004**
Monocyte count (cells/µl)	8	25	385	145 - 648	25	190	0 - 757.8	**0.002**
	10	25	378	100 - 600	25	297	0 - 812	0.137
	12	25	324	150 - 1176	25	348	0 - 1057	0.836
	14	25	354	93.1 - 696	25	360	93.4 - 762.3	0.812
	16	25	340	139 - 835	25	519	0 - 1420	0.306
	18	25	256	109 - 774	25	405	0 - 810	**0.006**
	20	25	308	0 - 940	25	206	0 - 525	**0.043**
	28	25	228	0 - 525	24	230	0 - 612	0.961
	36	25	177	0 - 408	24	230	0 - 924	0.053
	44	25	279	0 - 594	24	279	78.7 - 764	0.796
	52	25	254	0 - 676	24	150	0 - 768	**0.039**
Segmented neutrophil count (cells/µl)	8	25	3944	2546 - 7685	25	4526	2538 - 9699	0.316
	10	25	4875	2430 - 9699	25	5130	2433 - 11682	0.949
	12	25	5850	2457 - 8428	25	5170	2592 - 8909	0.631
	14	25	5895	2220 - 10561	25	4922	2895 - 7958.5	0.272
	16	25	6072	3046 - 10797	25	5588	3465 - 14570	0.599
	18	25	5976	2562 - 18576	25	5247	3052 - 10335	0.933
	20	25	4620	2987 - 10020	25	4440	2523 - 9780	0.265

	28	25	4560	2755 - 9072	24	5091	2485 - 9072	0.725
	36	25	3631	1683 - 9800	24	6120	2296 - 13629	**0.001**
	44				24		982.5 -	0.872
		25	3801	1215 - 6930		3681	15471	
	52				24		3448 –	**0.010**
		25	3910	1496 - 8450		4850	10430	
Hemoglobin (g/dl)	8	25	10	7.6 - 11.4	25	9.9	8.2 - 12.8	0.114
	10	25	8.9	7.0 - 10.7	25	9.4	8.1 - 10.8	0.273
	12	25	9.1	7.7 - 10.5	25	9.6	8.6 - 11.5	**0.021**
	14	25	9.5	6.9 - 11.5	25	10.1	8.6 - 13.3	**0.015**
	16	25	10	8.5 - 11.5	25	11.1	8.2 - 12.9	**0.000**
	18	25	10.1	8.8 - 12	25	11.3	9.7 - 13.3	**0.000**
	20	25	10.2	8.6 - 11.8	25	11.8	10 - 14.2	**0.000**
	28	25	12.1	10.8 - 14.7	24	14.0	11.7 - 15.5	**0.002**
	36	25	13.4	10.8 - 16.4	24	15.1	10.8 - 19.3	**0.008**
	44	25	14	11.1 - 20	24	13.2	11.5 - 16	0.311
	52	25	14	10.5 - 16.9	24	13.3	9.7 - 15.1	0.344
Hematocrit (%)	8	25	31.5	22.6 - 37.4	25	30.4	24.1 - 38.7	0.326
	10	25	28.4	23.1 - 33.8	25	29.6	24.5 - 33.9	0.440
	12	25	28.3	23.1 - 32.7	25	32 .0	26.4 - 38.4	**0.000**
	14	25	29.5	22.0 - 35.5	25	30 .0	26.0 - 35.2	0.954
	16	25	31.1	26.7 - 35.7	25	30.0	23.0 - 35.1	0.062
	18	25	32.0	29.1 - 39.7	25	32.2	26.8 - 37.2	0.608
	20	25	31.7	26.0 - 36.9	25	32.9	27.5 – 44.0	0.096
	28	25	35.5	31.3 – 44.0	24	38 .0	0 – 45.0	0.389
	36	25	39.4	31.3 - 47	24	36.9	29.3 - 46.1	0.153
	44	25	36.3	30.8 - 47.2	24	41.1	36.5 – 48.0	**0.001**
	52	25	38.5	27.3 - 44.1	24	40.45	30.8 - 44.4	**0.029**
Eosinophils (%)	8	25	8	4 - 12	25	13	7 - 18	**0.000**
	10	25	7	3 - 9	25	10	4 - 17	**0.000**
	12	25	6	3 - 15	25	9	3 - 13	**0.001**
	14	25	8	4 - 16	25	13	5 - 19	**0.000**
	16	25	8	3 - 15	25	8	4 - 18	0.326
	18	25	7	4 - 10	25	8	2 - 19	**0.006**
	20	25	7	1 - 12	25	11	6 - 17	**0.000**
	28	25	8	4 - 11	24	12	5 - 26	**0.000**
	36	25	9	1 - 21	24	8	5 - 14	0.810
	44	25	9	2 - 20	24	10	5 - 15	**0.037**
	52	25	6	2 - 13	24	7.5	3 - 23	**0.037**
Lymphocytes (%)	8	25	42	31 - 59	25	36	21 - 50	**0.003**
	10	25	42	32 - 51	25	40	18 - 61	0.157
	12	25	45	36 - 58	25	38	20 - 73	**0.007**
	14	25	41	23 - 82	25	37	24 - 55	0.093
	16	25	41	29 - 56	25	43	12 - 65	0.659
	18	25	42	18 - 80	25	38	24 - 59	0.115
	20	25	52	23 - 77	25	44	27 - 61	**0.020**
	28	25	45	29 - 61	24	43	31 - 63	0.456
	36	25	42	23 - 72	24	47	22 - 60	0.413
	44	25	48	23 - 74	24	49	10 - 69	0.947
	52	25	48	36 - 74	24	37	21 – 49	**0.000**
Monocytes (%)	8	25	4	1 - 7	25	2	0 - 9	**0.000**
	10	25	3	1 - 6	25	3	0 - 8	0.101

	12	25	3	1 - 6	25	3	0 - 7	0.520
	14	25	3	1 - 5	25	3	1 - 9	0.322
	16	25	2	1 - 6	25	4	0 - 10	0.059
	18	25	2	1 - 4	25	3	0 - 6	**0.004**
	20	25	3	0 - 4	25	2	0 - 5	0.303
	28	25	2	0 - 4	24	2	0 - 4	0.476
	36	25	2	0 - 5	24	2	0 - 6	0.608
	44	25	3	0 - 7	24	3	1 - 5	0.823
	52	25	3	0 - 5	24	2	0 – 4	**0.032**
Segmented neutrophils (%)	8	25	43	28 - 60	25	48	36 - 64	0.059
	10	25	46	38 - 62	25	48	29 - 74	0.845
	12	25	45	35 - 57	25	50	16 - 68	0.068
	14	25	45	12 - 67	25	46	31 - 58	0.785
	16	25	48	28 - 61	25	44	23 - 70	0.251
	18	25	48	14 - 72	25	47	28 - 65	0.726
	20	25	37	21 - 63	25	42	33 - 60	0.250
	28	25	45	29 - 63	24	41	24 - 56	0.240
	36	25	44	22 - 67	24	43	27 - 68	0.728
	44	25	41	15 - 66	24	41	15 - 81	0.656
	52	25	39	22 - 57	24	52	38 – 70	**0.000**
Platelet count (10^4 cells per µl)	8	25	23.1	8.59 - 52.2	25	27.80	18.10– 48.7	**0.034**
	10	25	24.2	15.0 - 47.0	25	24.4	13.60– 58.1	0.554
	12	25	25.6	14.9 - 54.5	25	25.7	12.2 – 50.3	0.857
	14	25	27.6	18.60- 73.6	25	36.0	18.70– 58.4	0.897
	16	25	31.5	18.1 - 60.9	25	39.6	20.6 – 56.5	0.163
	18	25	34.0	19.1 - 58.3	25	44.0	23.8 – 54.8	0.194
	20	25	38.8	17.8 - 91.0	25	49.5	17.8 – 67.9	**0.023**
	28	25	43.6	27.0 - 64.4	24	52.8	25.7 – 70.0	0.168
	36	25	29.2	23.5 - 109.6	24	45.0	24.6 – 55.2	0.455
	44	25	36.4	28.0 – 58.8	24	44.9	25.3 – 62.5	0.772
	52	25	52.6	22.7 – 86.8	24	39.9	20.6 – 48.7	0.064
Red blood cells (10^6 cells per µl)	8	25	6.72	5.02 – 8.30	25	6.90	5.90 – 8.92	0.352
	10	25	6.70	5.08 – 8.43	25	6.79	5.41 – 8.02	0.620
	12	25	7.01	4.65 – 8.48	25	7.51	6.33 – 9.08	**0.048**
	14	25	7.84	5.45 – 9.63	25	8.16	7.10 – 10.9	0.069
	16	25	8.10	6.66– 9.65	25	9.38	6.85 – 11.0	**0.000**
	18	25	8.51	7.23 – 10.2	25	9.59	8.32 – 11.6	**0.000**
	20	25	9.01	7.35 – 10.2	25	9.91	8.86 – 12.2	**0.000**
	28	25	10.2	8.52 – 13.1	24	11.40	10.2 – 14.0	**0.000**
	36	25	10.8	8.81 – 13.3	24	11.9	8.59 – 16.0	**0.005**
	44	25	11.4	8.77 – 15.4	24	10.2	9.36 – 12.7	0.070
	52	25	11.3	8.62 – 14.0	24	10.5	7.51 – 12.2	**0.010**
White blood cells (10^3 cells per µl)	8	25	9.18	5.68 – 15.1	25	9.4	5.64 – 15.9	0.963
	10	25	10.0	5.40 – 18.3	25	10.9	5.60 – 19.8	0.776
	12	25	12.7	6.64 – 20.3	25	11.2	7.44 – 16.2	0.130
	14	25	12.	6.75 – 20.9	25	10.9	7.090– 14.6	**0.015**
	16	25	13.8	8.96 – 18.3	25	13.7	8.25 – 23.5	0.888
	18	25	12.8	8.71 – 33.1	25	12.8	6.30 – 18.6	0.376
	20	25	13.3	7.95 – 23.5	25	10.5	6.29 – 17.7	**0.007**
	28	25	10.5	5.99 – 17.5	24	13.2	6.05 – 19.5	0.116
	36	25	9.0	5.10 – 17.5	24	12.2	6.30 – 23.5	**0.000**
	44	25	9.65	7.35 – 15.4	24	9.67	5.57 – 19.8	0.892

	52	25	10.1	6.80 – 16.9	24	9.95	5.87 – 19.2	0.883

Supplementary Table S2. Antibody titers in response to vaccinations shown as medians and ranges for control and supplemented kitten groups. *p*-values represent comparison between dietary groups at the sample timepoint, where values in bold denote statistical significance at $p < 0.05$.

Description	Week	Control			Supplemented			*p*-value
		n	Median	Range	n	Median	Range	
Feline calicivirus (FCV) antibody titer	8	25	90	90 - 90	25	90	90 - 90	1.000
	10	25	90	90 - 162	25	90	90 - 136	0.622
	12	25	156	90 - 1130	25	100	90 - 207	**0.039**
	14	25	2143	90 - 3806	25	2445	978 - 4588	**0.021**
	16	25	1294	282 - 2760	25	1101	604 - 3310	0.939
	18	25	962	260 - 1697	25	929	461 - 2155	0.272
	20	25	655	103 - 1590	25	684	352 - 2227	0.447
	28	25	717	181 - 1945	24	549	179 - 1945	0.324
	36	25	641	96.3 - 2430	24	608	90 - 2827	0.286
	44	25	614	109 - 2029	24	463	90 - 2178	**0.018**
	52	25	400	90 - 1552	24	297	109 - 2032	0.404
Chlamydia antibody titer	8	25	15	15 - 15	25	15	15 - 15	1.000
	10	25	15	15 - 40	25	15	15 - 34	0.689
	12	25	23	15 - 45	25	22	15 - 57	0.779
	14	25	26	15 - 51	25	36.9	27 - 97	**0.001**
	16	25	24.3	15 - 77	25	46.8	23 - 145	**0.001**
	18	25	30.6	15 - 180	25	101	28 - 234	**0.000**
	20	25	37.3	16 - 156	25	80.2	37 - 166	**0.000**
	28	25	100	15 - 532	24	146.5	62 - 356	**0.042**
	36	25	96.6	29 - 494	24	116	65 - 280	0.271
	44	25	137	51 - 425	24	108	35 - 260	**0.041**
	52	25	118	54 - 253	24	137	39- 248	0.783
Herpes virus antibody titer	8	25	50	50 - 50	25	50	50 - 50	1.000
	10	25	50	50 - 169	25	50	50 - 120	0.072
	12	25	50	50 - 818	25	50	50 - 205	0.887
	14	25	1137	50 - 1982	25	1168	475- 2200	0.346
	16	25	639	79- 1392	25	529	259 - 1420	0.657
	18	25	338	90 - 747	25	366	170 - 1860	0.177
	20	25	244	80 - 703	25	247	70 - 1040	0.342
	28	25	211	60 - 849	24	243	59 - 593	0.986
	36	25	178	50 - 713	24	132	50- 881	0.152
	44	25	190	50 - 908	24	122	50 - 900	0.119
	52	25	118	50 - 587	24	123	50 - 717	0.598
Panleukopenia virus antibody titer	8	25	300	300 - 300	25	300	300 - 300	1.000
	10	25	1883	300 – 3921	25	1606	300 - 5390	0.968
	12	25	17336	300 – 27,157	25	13093	300 – 24,898	0.097
	14	25	18170	5554 – 40,286	25	18244	1082 – 39,830	0.430
	16	25	22978	4829 – 40,066	25	23621	1015 – 46,601	0.942

	18	25	17238	2225 – 28,089	25	16749	730 – 37,258	0.785
	20	25	14690	1640 – 28,840	25	12074	722 – 23,593	0.343
	28	25	15866	1152 – 22,931	24	14552	2463 – 24,820	0.486
	36	25	18704	1445 – 27,199	24	14138	1824 – 20,807	**0.049**
	44	25	12903	1156 – 21,720	24	8353	1479 – 17,300	0.114
	52	25	11417	1082 – 29,071	24	15083	1866 – 26,194	0.903
Rabies virus antibody titer	12	25	0.12	0.12 - 32	25	0.12	0.12 - 0.12	0.447
	14	25	3.89	0.12 - 77	25	5.02	0.26 - 62.6	0.974
	16	25	25.9	0.12 - 92.6	25	20.8	7.07 - 91.2	0.936
	18	25	12.4	0.12 - 52.6	25	15.6	4.48 - 44.75	0.397
	20	25	6.05	0.12 - 24.6	25	5.47	1.36 - 25	0.778
	28	25	1.82	0.12 - 7.28	24	1.3	0.12 - 8.25	0.638
	36	25	0.98	0.13 - 4.45	24	0.89	0.12 - 5.06	0.488
	44	25	0.84	0.12 - 27.2	24	0.87	0.12 - 3.9	0.539
	52	25	0.72	0.12 - 11.5	24	0.8	0.12 - 3.95	0.605

Supplementary Table S3: Limit of quantification and limit of detection of all the cytokines tested.

Analyte	Limit of Quantification (pg/ml)	Limit of Detection (pg/ml)
sFas	13,7	4
Flt-3L	6,9	4
GM-CSF	13,7	6
IFN-g	68,6	43
IL-1B	34,3	14
IL-2	13,7	4
PDGF-BB	274,3	198
IL-12p40	13,7	9
IL-13	13,7	7
IL-4	68,6	30
IL-6	68,6	25
IL-8	13,7	7
KC	2,7	1
SDF-1	68,6	97
RANTES	2,7	1
SCF	68,6	48
MCP-1	137,2	164
TNF-a	13,7	7
IL-18	137,2	30
[redacted]		
IL-10	205 ng/ml	200 ng/ml

Supplementary Table S4: Cytokine expressions from the whole blood assays shown as medians and ranges for control and supplemented kitten groups. *p*-values represent comparison between dietary groups at the sample timepoint, where values in bold denote statistical significance at $p < 0.05$.

Week	W08									
Condition	PHA					RA				
Diet	Control		Supplemented		*p*-value	Control		Supplemented		*p*-value
	Median	Range	Median	Range		Median	Range	Median	Range	
FLT3L	0.04	-0.92 - 1.21	0.17	-0.25 - 1.03	**0.015**	0.25	-0.64 - 1.64	0.44	-0.5 - 1.16	0.271
GM-CSF	0	-0.2 - 2	0.15	-0.3 - 0.78	0.355	0.41	0 - 2.73	0.66	0 - 1.49	0.114
IL-12p40	0.94	-10.46 - 14.81	3.33	-0.82 - 10.43	**0.000**	3.02	-6.6 - 22.87	10.38	1.43 - 26.03	**0.000**
IL-13	0	-0.06 - 0.18	0.09	-0.04 - 0.92	**0.000**	0	0 - 0.86	0.18	-0.02 - 0.9	**0.000**
IL-18	0	-4.6 - 5.36	0	-5.4 - 5.92	**0.010**	0	-2.76 - 10.08	0	-2.22 - 7.43	0.135
IL-1β	4.49	-64.19 - 71.36	12.25	0 - 59.83	**0.003**	57.87	3.5 - 442.51	128.17	22.08 - 389.27	0.110
IL-2	0.29	-0.43 - 2.25	0.64	0.13 - 1.83	**0.002**	0.9	-0.03 - 11.72	0.89	0.21 - 3.39	0.393
IL-4	0	-1.47 - 3.04	1.56	-0.91 - 4.13	**0.002**	0	0 - 6.57	3.37	0 - 5.68	**0.000**
IL-6	3.3	0 - 24.99	18.12	6.19 - 45.99	**0.000**	11.8	0 - 29.65	31.37	11.57 - 86	**0.000**
IL-8	126.76	-6.72 - 840.18	193.9	18.96 - 535.26	0.088	346.42	24.49 - 1094.09	358.67	137.37 - 1029.08	0.376
IL-10	-0.36	-39.2 - 17.99	1.92	-30.95 - 14.7	0.071	2.74	-27.92 - 35.06	5.78	-37.78 - 23.58	0.462
KC	8.44	-7.58 - 30.69	30.74	3.1 - 91.69	**0.000**	16.66	-2.78 - 41.75	39.18	11.71 - 101	**0.001**
MCP-1	0	0 - 105.62	0	-28.54 - 99.84	0.483	0	0 - 207.9	37.07	0 - 109.88	0.095
PDGF-BB	1.32	-5.86 - 8.62	0	-0.47 - 6.81	0.123	2.34	5.86 - 32.53	1.15	-6.64 - 17.22	0.925
RANTES	20.39	-36.97 - 167.96	66.46	0.76 - 159.37	**0.001**	95.13	-33.17 - 211.39	137.49	32.9 - 217.99	**0.016**
TNF-α	0.51	-0.6 - 7.95	1.43	0.18 - 9.75	**0.000**	3.54	0.61 - 21.6	6.56	0.81 - 51.36	0.153

Week	W12									
Condition	PHA					RA				
Diet	Control		Supplemented		*p*-value	Control		Supplemented		*p*-value
	Median	Range	Median	Range		Median	Range	Median	Range	
FLT3L	0.06	-0.09 - 0.42	0.13	-0.46 - 0.55	0.967	0.07	-0.21 - 0.7	0	-0.58 - 1.26	0.182
GM-CSF	0	-0.39 - 11.88	0	-2.22 - 4.51	0.481	0.48	0 - 11.96	0.98	-1.16 - 7.21	0.520
IL-12p40	1.03	-1.27 - 10.49	2.02	-8.56 - 7.87	0.256	2.05	-0.7 - 10.64	3.57	-1.72 - 14.22	0.275
IL-13	0	0 - 0.43	0	0 - 0.05	0.197	0	0 - 0.89	0	0 - 0.22	0.299
IL-18	0.53	-1.9 - 4.74	0.39	-2.81 - 5.29	0.773	1.01	-3.89 - 11.38	0.77	-4.4 - 4.74	0.766
IL-1β	2.85	-17.97 - 53.11	5.74	-13.65 - 28.16	0.160	46.35	-2.92 - 109.88	42.24	-6.41 - 207.8	0.735
IL-2	0.19	-0.24 - 0.76	0.31	-0.66 - 1.54	0.175	0.43	-0.2 - 1.88	0.55	-0.56 - 2.38	0.710
IL-4	0.49	-1.7 - 4.08	0	0 - 4.63	0.946	1.06	-0.08 - 9.12	0.88	0 - 4.63	0.710
IL-6	1.67	0 - 16.57	5.36	0 - 33.77	**0.001**	6.97	0 - 27.77	12.92	3.6 - 39.51	**0.002**
IL-8	105.76	-29.15 - 413.28	72.05	-33.85 - 1043.39	0.638	195.9	18.88 - 552.64	82.91	-26.48 - 636.74	**0.003**
IL-10	0.03	-48.1 - 10.2	0.91	-13.1 - 57.05	0.153	2.51	-7.07 - 25.93	3.37	-5.71 - 19.65	0.235

KC	11.1	-3.44 - 28.98	11.36	0.58 - 22.09	0.994	14.58	-4.3 - 35.25	7.4	-10.64 - 22.76	**0.007**
MCP-1	10.86	-23.67 - 65.01	25.22	-29.97 - 84.54	0.547	27.83	-5.67 - 120.79	23.9	-46.86 - 107.65	0.403
PDGF-BB	0	-9.77 - 17.71	0	-12.61 - 5.19	**0.032**	3.09	0 - 30.59	0	-17.22 - 7.78	**0.000**
RANTES	47.48	-76.81 - 165.58	44.52	-27.91 - 100.25	0.994	68.07	-9.12 - 146.72	61.16	1.38 - 116.61	0.486
TNF-α	0.42	-3.4 - 3.12	0.65	-3.21 - 3.05	0.293	1.82	-1.45 - 14.8	2.76	-1.74 - 8.75	0.222

**Week**	**W16**									
**Condition**	**PHA**					**RA**				
**Diet**	**Control**		**Supplemented**		***p*-value**	**Control**		**Supplemented**		***p*-value**
	**Median**	**Range**	**Median**	**Range**		**Median**	**Range**	**Median**	**Range**	
FLT3L	0.06	-0.23 - 0.46	0.03	-1.25 - 1.79	0.328	-0.01	-0.47 - 0.36	0.02	-1.37 - 0.44	0.512
GM-CSF	0	0 - 2.01	0.15	-0.35 - 2.25	0.627	0.39	-0.11 - 3.9	0.56	-0.35 - 4.11	0.359
IL-12p40	0.29	-1.16 - 2.12	0.52	-1.59 - 5.01	0.354	0.88	-1.7 - 4.38	1.57	-0.85 - 10.68	**0.024**
IL-13	0.03	-0.12 - 0.32	0.07	-1.22 - 1.76	0.709	0	-0.32 - 0.78	0.04	-1.03 - 0.63	**0.003**
IL-18	0.58	-2.76 - 6.54	0.74	-17.6 - 35.27	0.845	0.31	-6.5 - 7.87	1.61	-21 - 15	0.084
IL-1β	2.88	-9.63 - 27.5	3.58	-11.34 - 22.5	0.976	10.97	-2.66 - 58.83	21.74	-11.34 - 70.58	0.969
IL-2	0.23	-1.86 - 1.44	0.26	-0.45 - 2.37	0.686	0.5	-3.04 - 11.11	0.34	-0.48 - 1.18	0.100
IL-4	1.02	-5.83 - 8.58	1.11	-35.12 - 41.09	0.441	0.23	-11.49 - 6.71	1.02	-33.12 - 11.61	0.076
IL-6	1.92	-1.47 - 14.16	2.95	-12.93 - 13.68	0.814	2.62	-2.46 - 13.46	4.71	-14.62 - 16.82	0.096
IL-8	3.99	-9.56 - 84.65	16.26	-38.86 - 105.29	0.085	13.74	-20.82 - 68.95	25.32	-46.29 - 268.88	0.075
IL-10	0.99	-121.93 - 259.3	0	-48.57 - 13.06	0.303	4.25	-120.67 - 244.53	3.03	-32.33 - 49.04	0.422
KC	6.56	-1.77 - 15.84	12.81	-10.59 - 42.78	**0.038**	9.35	-5.04 - 20.97	13.27	-12.41 - 62.78	0.092
MCP-1	10.77	-21.14 - 35.59	15.88	-205.13 - 425.95	0.952	1.43	-53.41 - 93.25	21.79	-183.23 - 257.91	**0.010**
PDGF-BB	0	-11.27 - 11.89	0.4	-4.39 - 11.23	0.201	0	-11.27 - 17.65	0.93	-6.94 - 15.69	**0.016**
RANTES	10.99	-12.75 - 40.15	16.55	-18.42 - 115.09	0.171	16.64	1.51 - 58.38	33.62	-22.57 - 125.89	0.081
TNF-α	0.86	-1.14 - 2.29	0.65	-9.77 - 12.88	0.612	0.9	-2.56 - 8.73	1.63	-9.52 - 12.23	0.050

**Week**	**W20**									
**Condition**	**PHA**					**RA**				
**Diet**	**Control**		**Supplemented**		***p*-value**	**Control**		**Supplemented**		***p*-value**
	**Median**	**Range**	**Median**	**Range**		**Median**	**Range**	**Median**	**Range**	
FLT3L	0.08	-0.32 - 0.48	0.13	-0.08 - 0.97	0.172	0.02	-0.91 - 0.37	0.02	-0.48 - 1.68	0.543
GM-CSF	0	0 - 0.84	0	0 - 0.62	0.118	0.17	0 - 1.69	0	0 - 2.76	0.407
IL-12p40	0.38	-0.77 - 2.77	0.89	-1.07 - 4.64	0.070	0.82	-0.92 - 5.07	2.98	-0.77 - 19.22	**0.010**
IL-13	0	0 - 0.39	0.03	0 - 2.16	0.200	0	-0.65 - 0.28	0	-0.37 - 2.01	0.694
IL-18	0.68	-4.2 - 4.23	1.63	-1.81 - 9.88	0.242	0.26	-4.35 - 2.91	0	-4.14 - 25.63	0.650
IL-1β	5.39	-16.09 - 34.59	5.22	-13.1 - 10.4	0.291	13.52	-0.21 - 96.42	21.94	-3.31 - 43.98	0.224
IL-2	0	0 - 0.83	0.21	-0.43 - 1.35	0.416	0.88	0 - 7.42	0.54	-0.37 - 2.47	0.701
IL-4	2.54	-12.38 - 9.71	0	0 - 9.19	**0.035**	0.78	-12.38 - 6.13	0	-6.75 - 23.16	**0.030**
IL-6	3.37	-4.61 - 13.5	0	0 - 8.13	**0.024**	5.23	-1.69 - 11.07	0	-2.95 - 14.35	0.139

IL-8	13.6	-24.33 - 67.38	70.84	-36.76 - 377.11	**0.000**	31.75	-6.26 - 115.39	105.73	-26.29 - 1303.56	**0.000**
IL-10	3.29	-64.94 - 290.23	0.78	-31.53 - 18.9	0.288	3.31	-42.67 - 34.22	1.84	-35.86 - 20.19	0.950
KC	6.37	0.76 - 21.39	15.22	-1.79 - 41.09	**0.006**	11.27	0.86 - 30.8	14.55	-2.4 - 55.15	0.176
MCP-1	22.96	-115.18 - 83.83	20.3	0 - 166.01	0.972	7.45	-106.94 - 53.28	0.19	-44.89 - 293.88	0.660
PDGF-BB	0	-7.35 - 9.81	3.31	-0.96 - 15.69	**0.000**	0	-7.35 - 9.81	4.57	-1.25 - 17.03	**0.000**
RANTES	14.04	-11.11 - 80.89	15	-4.32 - 95.97	0.191	25.5	2.5 - 85.67	55.74	6.26 - 192.24	**0.007**
TNF-α	0.79	-3.73 - 2.93	0.32	0 - 3.31	0.713	0.88	-3.11 - 2.45	0.88	-1.93 - 7.48	0.575

Supplementary Table S5: Relative bacterial phyla distribution (%) in control and supplemented groups at all timepoints sampled (weeks 4, 8, 12, 16, 20, 28, 36 and 52). CTL: control group; SUP: supplemented group. W: week.

**Phylum**	**CTL_W4**	**CTL_W8**	**CTL_W12**	**CTL_W16**	**CTL_W20**	**CTL_W28**	**CTL_W36**	**CTL_W44**	**CTL_W52**
Actinobacteria	5.27	2.07	1.18	1.99	3.21	3.94	5.72	4.52	4.61
Aquificae	0	0	0	0	0	0	0	0	0
Bacteroidetes	19.74	12.2	11.2	15.18	13.55	16.6	22.58	19.55	17.78
Chordata	0	0	0	0	0	0	0	0.01	0
Firmicutes	56.36	60.62	69.19	68.56	67.93	64.5	60.34	59.87	61.72
Fusobacteria	7.08	11.95	6.81	4.51	5.97	7.66	3.72	6.67	5.11
Proteobacteria	11.55	13.16	11.62	9.76	9.34	7.29	7.64	9.38	10.78
Verrucomicrobia	0	0	0	0	0	0	0	0	0

**Phylum**	**SUP_W4**	**SUP_W8**	**SUP_W12**	**SUP_W16**	**SUP_W20**	**SUP_W28**	**SUP_W36**	**SUP_W44**	**SUP_W52**
Actinobacteria	3.18	1.86	1.24	2.38	3.1	2.75	3.96	4.41	5.02
Aquificae	0	0	0	0	0	0	0	0	0
Bacteroidetes	21.16	16.29	14.14	20.43	25.41	24.43	26.25	27.36	27.22
Chordata	0	0	0	0	0	0	0	0	0
Firmicutes	55.9	61.83	71.82	65.65	60.73	60.28	58.68	55.67	54.56
Fusobacteria	10.84	11.07	2.95	3.14	2.23	2.16	1.99	2.29	3.35
Proteobacteria	8.91	8.95	9.82	8.4	8.54	10.36	9.12	10.28	9.87
Verrucomicrobia	0	0	0.02	0	0	0	0	0	0

Supplementary Table S6: PERMANOVA comparisons of beta diversity of all samples. CTL: control group; SUP: supplemented group.

Contrasts	factors	Df	SumOfSqs	R^2	F	Pr(>F)
CTL_W04_vs_CTL_W08	Model	25	4.83	0.62	1.56	0.00
	Residual	24	2.98	0.38	NA	NA
	Total	49	7.80	1.00	NA	NA
CTL_W08_vs_CTL_W12	Model	25	2.76	0.56	1.20	0.00
	Residual	24	2.20	0.44	NA	NA
	Total	49	4.97	1.00	NA	NA
CTL_W12_vs_CTL_W16	Model	25	2.75	0.64	1.68	0.00
	Residual	24	1.57	0.36	NA	NA
	Total	49	4.32	1.00	NA	NA
CTL_W16_vs_CTL_W20	Model	25	2.75	0.63	1.63	0.01
	Residual	24	1.62	0.37	NA	NA
	Total	49	4.38	1.00	NA	NA
CTL_W20_vs_CTL_W28	Model	25	3.37	0.67	1.99	0.00
	Residual	24	1.62	0.33	NA	NA
	Total	49	4.99	1.00	NA	NA
CTL_W28_vs_CTL_W36	Model	25	3.17	0.64	1.71	0.00
	Residual	24	1.77	0.36	NA	NA
	Total	49	4.94	1.00	NA	NA
CTL_W36_vs_CTL_W44	Model	25	3.83	0.68	2.02	0.00
	Residual	24	1.82	0.32	NA	NA
	Total	49	5.65	1.00	NA	NA
CTL_W44_vs_CTL_W52	Model	25	3.52	0.60	1.47	0.08
	Residual	24	2.30	0.40	NA	NA
	Total	49	5.82	1.00	NA	NA
	Model	1	0.50	0.06	3.30	0.003
	Residual	48	7.33	0.94	NA	NA
CTL_W04_vs_SUP_W04	Total	49	7.83	1.00	NA	NA
CTL_W08_vs_SUP_W08	Model	1	0.33	0.07	3.49	0.002
	Residual	48	4.59	0.93	NA	NA
	Total	49	4.93	1.00	NA	NA
CTL_W12_vs_SUP_W12	Model	1	0.39	0.09	4.59	0.001
	Residual	48	4.10	0.91	NA	NA
	Total	49	4.49	1.00	NA	NA
CTL_W16_vs_SUP_W16	Model	1	0.77	0.16	8.91	0.001
	Residual	48	4.16	0.84	NA	NA
	Total	49	4.93	1.00	NA	NA
CTL_W20_vs_SUP_W20	Model	1	1.30	0.24	15.29	0.001
	Residual	48	4.09	0.76	NA	NA
	Total	49	5.39	1.00	NA	NA

CTL_W28_vs_SUP_W28	Model	1	1.00	0.20	12.04	0.001
	Residual	47	3.89	0.80	NA	NA
	Total	48	4.89	1.00	NA	NA
CTL_W36_vs_SUP_W36	Model	1	0.73	0.13	7.16	0.001
	Residual	47	4.81	0.87	NA	NA
	Total	48	5.55	1.00	NA	NA
CTL_W44_vs_SUP_W44	Model	1	0.55	0.10	5.26	0.001
	Residual	47	4.94	0.90	NA	NA
	Total	48	5.49	1.00	NA	NA
CTL_W52_vs_SUP_W52	Model	1	0.88	0.15	8.54	0.001
	Residual	47	4.83	0.85	NA	NA
	Total	48	5.71	1.00	NA	NA
SUP_W04_vs_SUP_W08	Model	25	3.46	0.61	1.52	0.04
	Residual	24	2.18	0.39	NA	NA
	Total	49	5.65	1.00	NA	NA
SUP_W08_vs_SUP_W12	Model	25	3.50	0.66	1.85	0.00
	Residual	24	1.81	0.34	NA	NA
	Total	49	5.31	1.00	NA	NA
SUP_W12_vs_SUP_W16	Model	25	2.91	0.63	1.61	0.01
	Residual	24	1.74	0.37	NA	NA
	Total	49	4.65	1.00	NA	NA
SUP_W16_vs_SUP_W20	Model	25	3.03	0.68	2.07	0.00
	Residual	24	1.40	0.32	NA	NA
	Total	49	4.43	1.00	NA	NA
SUP_W20_vs_SUP_W28	Model	1	0.19	0.05	2.50	0.03
	Residual	47	3.57	0.95	NA	NA
	Total	48	3.76	1.00	NA	NA
SUP_W28_vs_SUP_W36	Model	24	2.89	0.70	2.22	0.15
	Residual	23	1.25	0.30	NA	NA
	Total	47	4.14	1.00	NA	NA
SUP_W36_vs_SUP_W44	Model	24	3.37	0.75	2.80	0.03
	Residual	23	1.15	0.25	NA	NA
	Total	47	4.52	1.00	NA	NA
SUP_W44_vs_SUP_W52	Model	24	3.13	0.75	2.82	0.27
	Residual	23	1.07	0.25	NA	NA
	Total	47	4.20	1.00	NA	NA

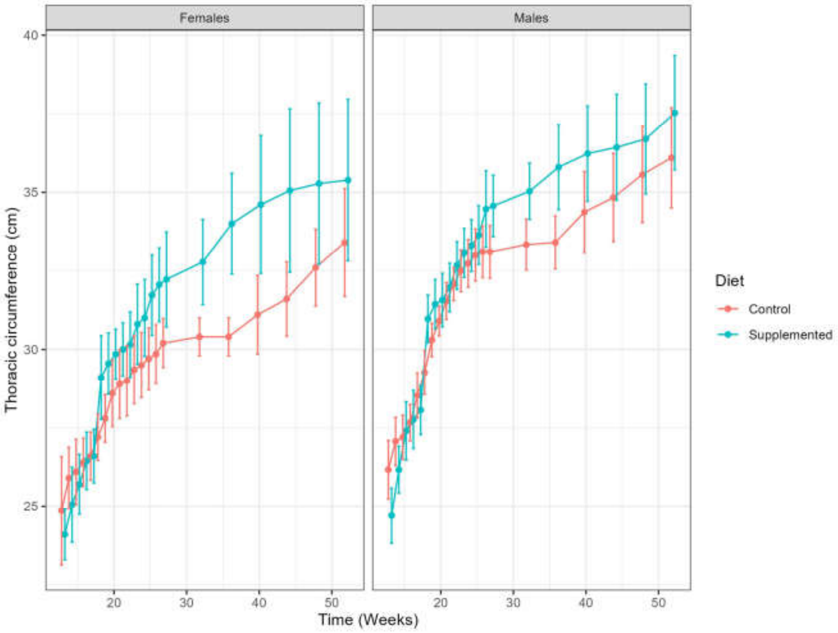

Supplementary Figure S1. Changes in mean thoracic circumference (cm) in females and males from 12 to 52 weeks of age, showing control (red) and supplemented (blue) kittens. Values shown are group means and ranges. Note the upward trajectory from around 30 and 40 weeks in supplemented and control females respectively and at around 26 and 36 weeks in supplemented and control males, corresponding with time of neutering.

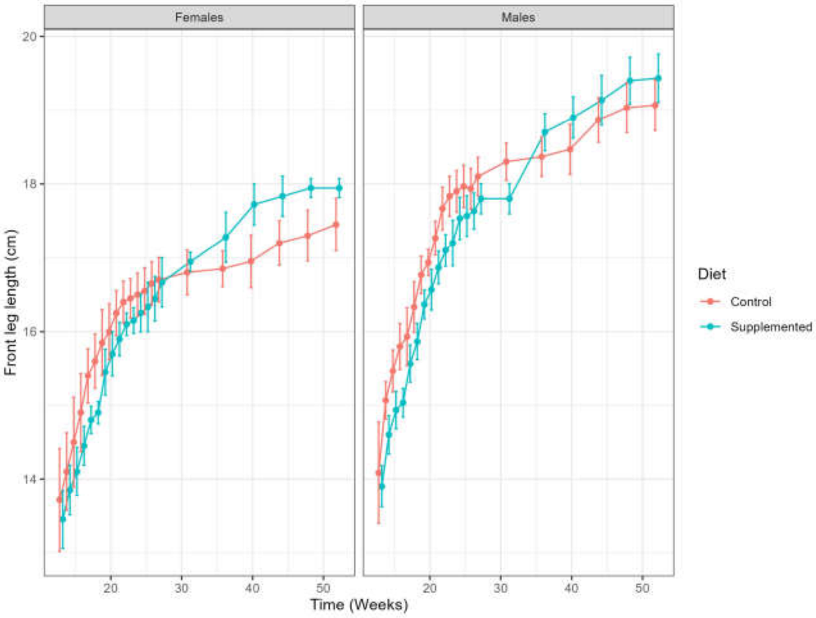

Supplementary Figure S2. Changes in mean front leg length (cm) in females and males from 12 to 52 weeks of age, showing control (red) and supplemented (blue) kittens. Values shown are group means and ranges. Note the upward trajectory from around 30 and 40 weeks in supplemented and control females respectively and at around 32 weeks in supplemented males, corresponding with time of neutering.

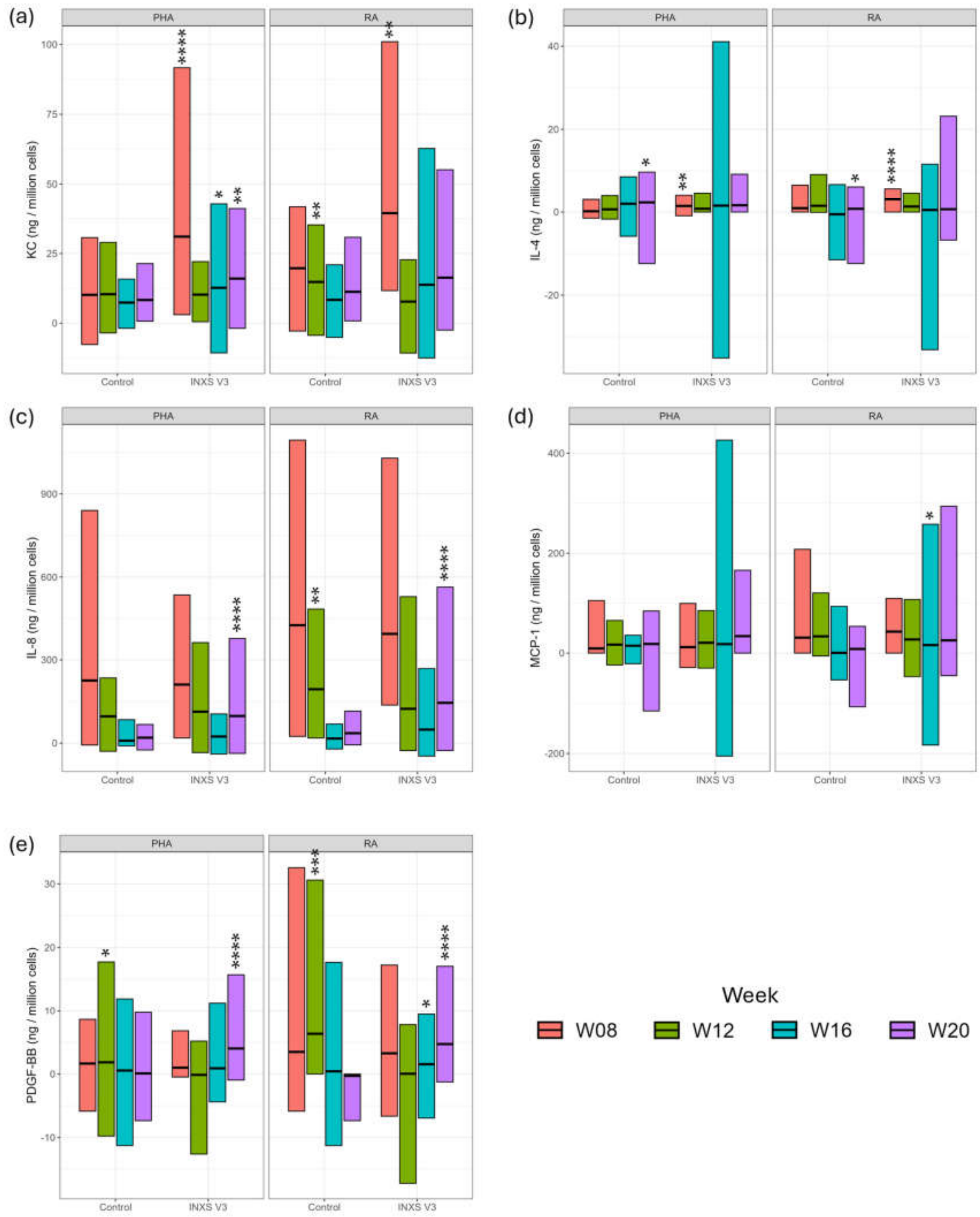

Supplementary Figure S3: Serum cytokine analysis showing a weaker response to RA stimulation at week 8 (red), week 12 (green), week 16 (blue) and week 20 (purple) in response to PHA stimulation (positive control) and RA stimulation in control and treatment groups. Data are presented as means and ranges **(a)** KC; **(b)** IL-4; **(c)** IL-8; **(d)** MCP-1; **(e)** PDGF-BB. * significant difference at $p < 0.05$; ** significant difference at $p < 0.01$; *** significant difference at $p < 0.001$; **** significant difference at $p < 0.0001$.

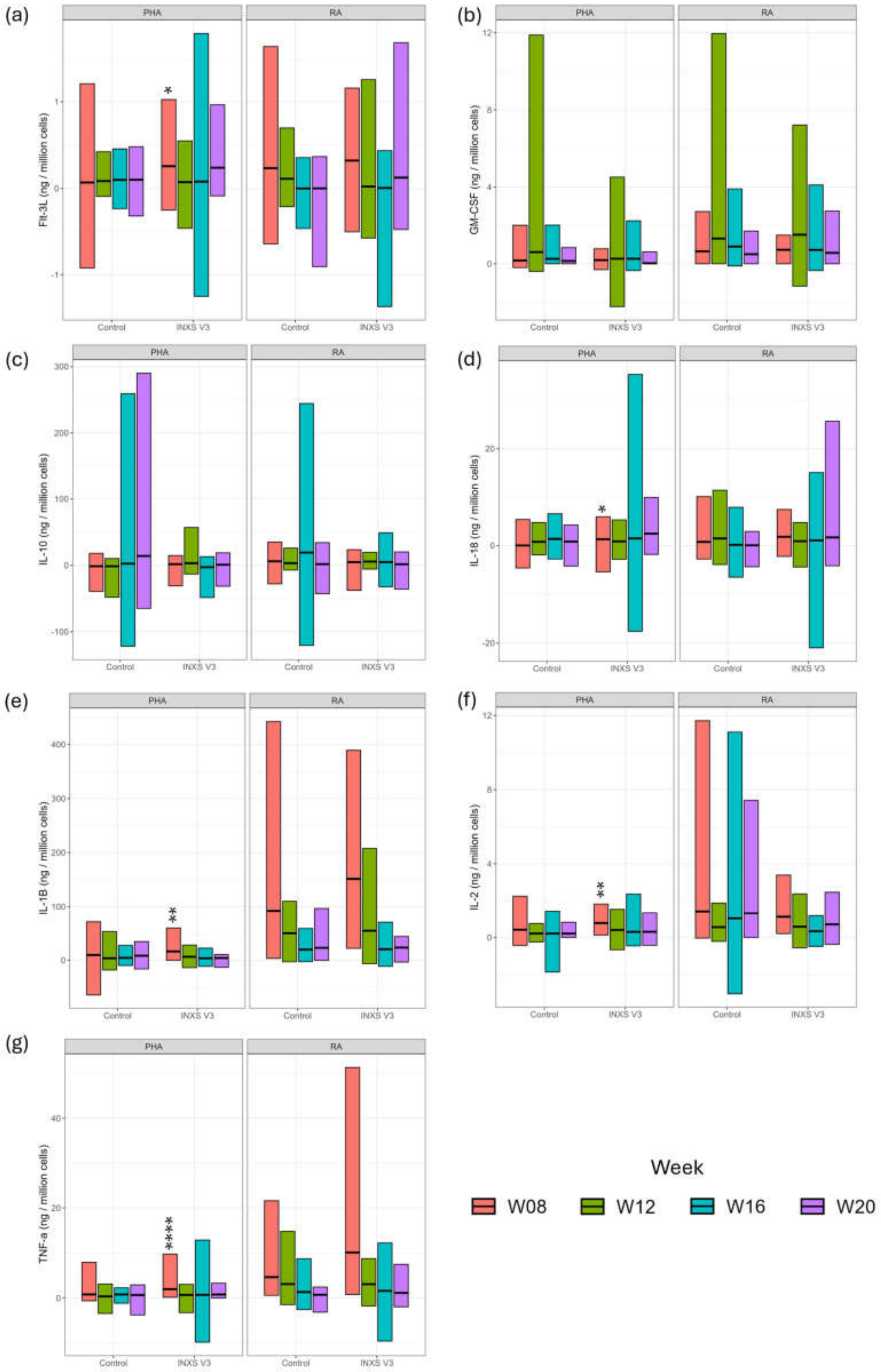

Supplementary Figure S4: Serum cytokine analysis showing no response after RA stimulation at week 8 (red), week 12 (green), week 16 (blue) and week 20 (purple) in response to PHA stimulation (positive control) and RA stimulation in control and treatment groups. Data are presented as means and ranges **(a)** Flt-3L; **(b)** GM-CSF; **(c)** IL-10; **(d)** IL-18; **(e)** IL-1β; **(f)** IL-2; **(g)** TNF-α. * significant difference at $p < 0.05$; ** significant difference at $p < 0.01$; *** significant difference at $p < 0.001$; **** significant difference at $p < 0.0001$.

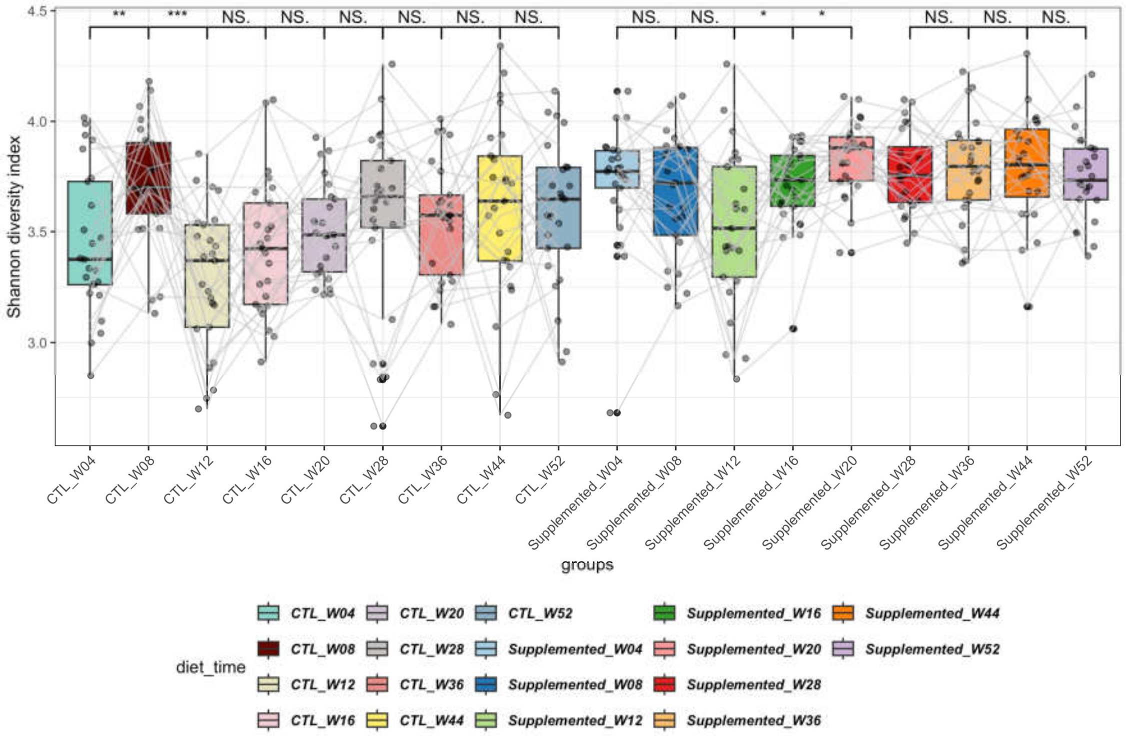

Supplementary Figure S5: Alpha diversity of samples across diet and time, compared by time, metrics: Shannon index. Grey lines connect each individual at each time point. NS: non-significant; * significant difference at $p < 0.05$.

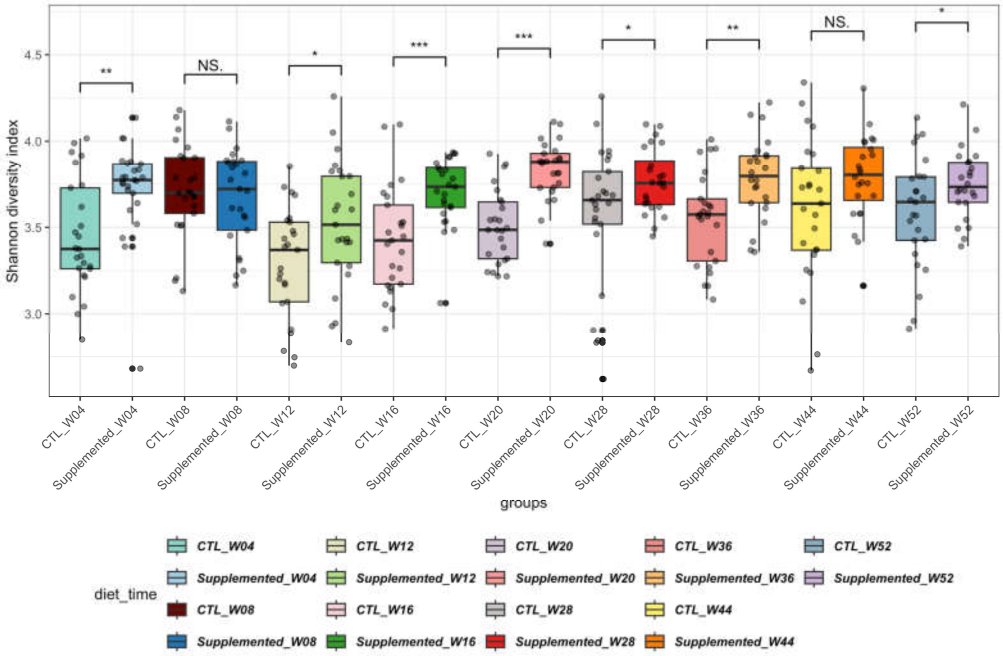

Supplementary Figure S6: Alpha diversity of samples across diet and time, compared by diet, metrics: Shannon index. NS: non-significant; * $p < 0.05$; ** $p < 0.01$; *** $p < 0.001$.

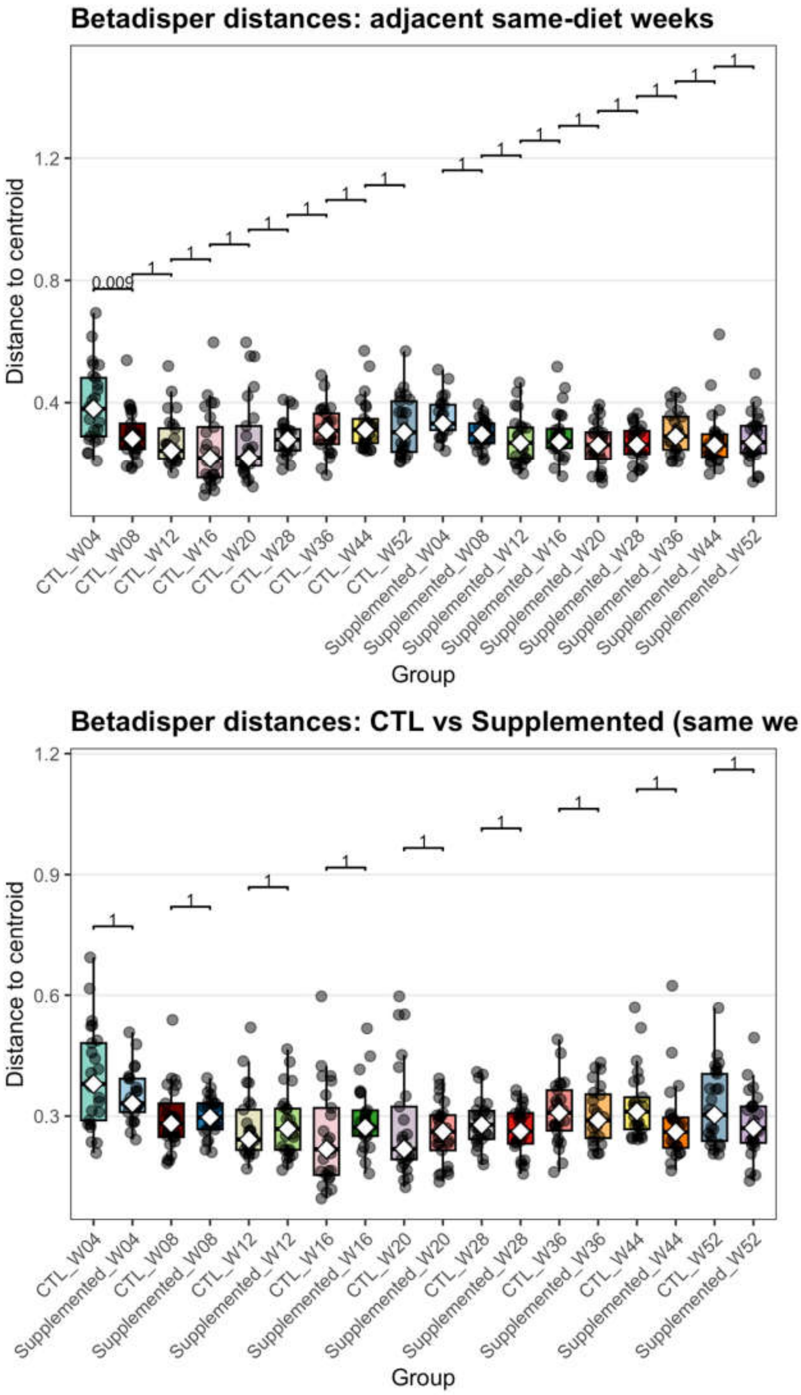

Supplementary Figure S7: Boxplot of the beta dispersion analysis. Boxplots show the distribution of Bray–Curtis distances from samples to their group centroid (betadisper) for all diet–time categories, summarizing multivariate dispersion (beta diversity variability) across groups.
